# “Can’t live willingly”: A thematic synthesis of qualitative evidence exploring how early marriage and early pregnancy affect experiences of pregnancy in South Asia

**DOI:** 10.1371/journal.pgph.0002279

**Published:** 2023-10-23

**Authors:** Faith A. Miller, Sophiya Dulal, Anjana Rai, Lu Gram, Helen Harris-Fry, Naomi M. Saville

**Affiliations:** 1 Institute for Global Health, University College London, London, United Kingdom; 2 School of Health Sciences, Western Sydney University, Sydney, Australia; 3 School of Public Health and Social Work, Queensland University of Technology, Brisbane, Australia; 4 Department of Population Health, The London School of Hygiene & Tropical Medicine, London, United Kingdom; The University of Texas Health Science Center at Houston School of Public Health - San Antonio Campus, UNITED STATES

## Abstract

In South Asia, early marriage has been associated with a range of adverse outcomes during pregnancy and infancy. This may partly be explained by early marriage leading to a younger maternal age, however it remains unclear which other factors are involved. This review aimed to synthesise the qualitative evidence on experiences of pregnancy following early marriage or early pregnancy in South Asia, to inform our understanding of the mechanisms between early marriage and adverse pregnancy outcomes. We searched MEDLINE, EMBASE, Scopus, Global Index Medicus, CINAHL, PsycINFO, Web of Science, and grey literature on 29/11/2022 to identify papers on experiences of pregnancy among those who married or became pregnant early in South Asia (PROSPERO registration number: CRD42022304336, funded by an MRC doctoral training grant). Seventy-nine papers from six countries were included after screening. We appraised study quality using an adapted version of the Critical Appraisal Skills Programme tool for qualitative research. Reporting of reflexivity and theoretical underpinnings was poor. We synthesised findings thematically, presenting themes alongside illustrative quotes. We categorised poor pregnancy experiences into: care-seeking challenges, mental health difficulties, and poor nutritional status. We identified eight inter-connected themes: restrictive social hierarchies within households, earning social position, disrupted education, social isolation, increased likelihood of and vulnerability to abuse, shaming of pregnant women, normalisation of risk among younger women, and burdensome workloads. Socioeconomic position and caste/ethnic group also intersected with early marriage to shape experiences during pregnancy. While we found differences between regions, the heterogeneity of the included studies limits our ability to draw conclusions across regions. Pregnancy experiences are largely determined by social hierarchies and the quality of relationships within and outside of the household. These factors limit the potential for individual factors, such as education and empowerment, to improve experiences of pregnancy for girls married early.

## Introduction

### Early marriage

Early marriage is defined as a marriage or informal union in which one or both partners is below the age of 18 years [1]. While rates of early marriage have decreased globally from an estimated 25% to 20% among girls since 2010, it remains common in South Asia, where around one third of marriages occur during childhood [2]. Early marriage has been associated with adverse maternal and infant health outcomes such as pre-eclampsia, antepartum haemorrhage, delivering a low birthweight baby, preterm delivery, and infant mortality [3–6]. For some outcomes, this may be partly explained by an increased likelihood of becoming pregnant at a young age, however there are also independent effects of early marriage, although the mechanisms are poorly understood [5, 7, 8]. Understanding the mechanisms between early marriage and adverse maternal and infant health is critical to understand what support is needed to improve the health and wellbeing of girls married early [9].

Early marriage is intimately connected with local cultural and gender norms which simultaneously drive the practice of early marriage and shape experiences [10, 11]. In alignment with these norms, households tend to have hierarchies relating to gender and age, meaning young married girls are often discriminated against [12, 13]. In South Asia, early marriage has been associated with interrupted education, restricted mobility outside of the home, social isolation, and limited decision-making [14–17]. This can negatively impact care-seeking during pregnancy [18]. Girls married early are also more vulnerable to gender-based violence within their marital homes [19]. Young brides in South Asia report lower uptake of contraception and higher rates of pregnancy termination than those marrying in adulthood, highlighting the adverse reproductive health sequelae [20, 21]. Inter-connected with each of these experiences is mental health; while large studies are lacking, there is increasing evidence that early marriage has negative mental health consequences [22, 23]. This highlights the complexity which must be considered when exploring outcomes from early marriage.

Over recent decades, the evidence exploring consequences of early marriage has grown. Quantitative reviews support an association between early marriage and a range of adverse reproductive and maternal health outcomes, but highlight the dearth of evidence exploring the mechanisms involved [24–26]. Qualitative studies are well suited to explore such mechanisms, as they centre upon the lived experiences, motivations, and behaviours of those affected [27]. South Asia is a culturally diverse region, represented by its linguistic, religious, ethnic, and geographical diversity between and within countries [28, 29]. Furthermore, differing rates of socioeconomic development and demographic transitions in different countries and sub-national regions mean there are differences in the health and social challenges faced across South Asia [30]. However, similar social structures, norms, and values persist across South Asia which affect experiences of early marriage [23–25, 31]. Therefore, summaries of context-specific evidence are required. Seeking to address this gap, this review aims to synthesise the qualitative literature from South Asia on how early marriage and early pregnancy interact in shaping pregnancy experiences. We aim to provide insight on the mechanisms through which early marriage may affect maternal health outcomes.

## Methods

The review protocol was registered on PROSPERO on 10/02/2022 (registration number: CRD42022304336) [32].

### Eligibility criteria

We used the PICOS framework to outline eligibility criteria, summarised in Table 1.

**Table 1:** PICOS framework to determine eligibility.

Participants	Study conducted in at least one South Asian country (According to the UN classification: Afghanistan, Bangladesh, Bhutan, India, Maldives, Nepal, Pakistan, and Sri Lanka [33]) Participants with first- or second-hand experience of pregnancy or childbirth (current or previous); may include partners, parents, parents-in-law, healthcare providers
Intervention/Exposure	Marriage or pregnancy at an early age as characterised by the authors (including but not limited to <18 years, ‘adolescent’, ‘early’, ‘young’)
Comparison	Marriage or pregnancy at a later age *Reference to a comparator group may be implied*
Outcome	Experiences of pregnancy or childbirth, including but not limited to nutrition, psychosocial health, care-seeking, and family relationships
Study type	Studies reporting primary data with a qualitative methodology, including mixed-methods studies with a qualitative component *Brief answers to open-ended survey questions are not considered qualitative*

### Search strategy

Searches were conducted by FM using Ovid (MEDLINE), EMBASE, Scopus, Global Index Medicus, CINAHL, PsycINFO, Web of Science, and the ProQuest Dissertation & Theses Global database. Searches were run on 31/01/2022 and updated on 29/11/2022, using a combination of free text terms, Medical Subject Heading terms, and database-specific limiters, with no language or date restrictions (S1 Appendix). Forward and backward citation searching was undertaken to capture resources citing or being cited by the included literature [34]. FM searched websites of relevant organisations using the terms ‘Marriage’ and ‘Pregnancy’ (S1 Appendix).

### Study selection

Two authors screened 10% of titles and abstracts (5% FM & HHF, 5% FM & NS) and disagreements were resolved by discussion. As authors agreed in >90% of papers, FM proceeded to screen the remaining titles and abstracts independently. 10% of full-texts were then screened by two authors (5% FM & HHF, 5% FM & NS), with disagreements resolved by discussion. As authors agreed in >90% of papers, FM screened the remaining full-texts.

### Quality appraisal

We appraised study quality using the Critical Appraisal Skills Programme (CASP) tool for qualitative research, which consists of 10 questions which help appraise the strengths and limitations of qualitative methodology [35]. We selected the CASP tool for its useability and focus on contextualising study findings, however we included an additional question on theoretical underpinnings because the tool has been criticised for not being sensitive to the theoretical validity of studies [35–37]. FM and LG appraised a subset of studies together (10%; 7 papers) before FM appraised the remaining studies independently. Studies were rated ‘High’, ‘Medium’, or ‘Low’ relevance, according to the quality of reporting and relevance to the review question.

### Thematic synthesis

There are a range of approaches to qualitative synthesis, which differ in the way they identify studies for inclusion, examine similarities and differences between studies, appraise study quality, and go beyond the primary studies to generate additional understanding [38, 39]. Thematic syntheses are similar to meta-ethnography in the way they generate higher level analytical themes from descriptive themes identified within the primary studies, however they use a more clearly defined approach to searching and quality appraisal [40, 41]. Thematic syntheses are well suited to hypothesis generation and synthesising findings from studies spanning multiple disciplines and paradigms, making it well-suited to the aims of this review [42, 43]. However, this approach has been criticised for decontextualising findings, therefore efforts must be made to consider how differences between contexts may explain contradictions between studies [42].

We extracted data on item characteristics, research aims, methods, and findings and entered these into an Excel spreadsheet. We uploaded papers to NVivo 12 for synthesis of the ‘results/findings’ section of each paper. In two case study papers author interpretation took place in the ‘discussion’ and therefore the ‘discussion’ was also extracted for synthesis.

We synthesised findings thematically as follows: i) coding the results of primary studies line-by-line (according to their context), ii) comparing codes between studies (considering similarities and differences), and iii) developing analytical themes (focusing on the review aims to generate a higher-level interpretation, while considering the context of each study to prevent de-contextualisation) [40]. When synthesising findings we sought out contradictory evidence to challenge the themes we were developing.

AR, FM & SD coded ‘High’-relevance studies inductively, each coding independently and developing initial themes together through discussion and comparison. FM then deductively coded ‘medium’- and ‘low’-relevance studies, updating the themes iteratively in discussion with co-authors. This research was primarily undertaken by FM as part of her PhD. As a non-South Asian woman, FM has worked closely with researchers from South Asia (AR and SD) for this review to contextualise findings.

## Results

**Fig 1:**
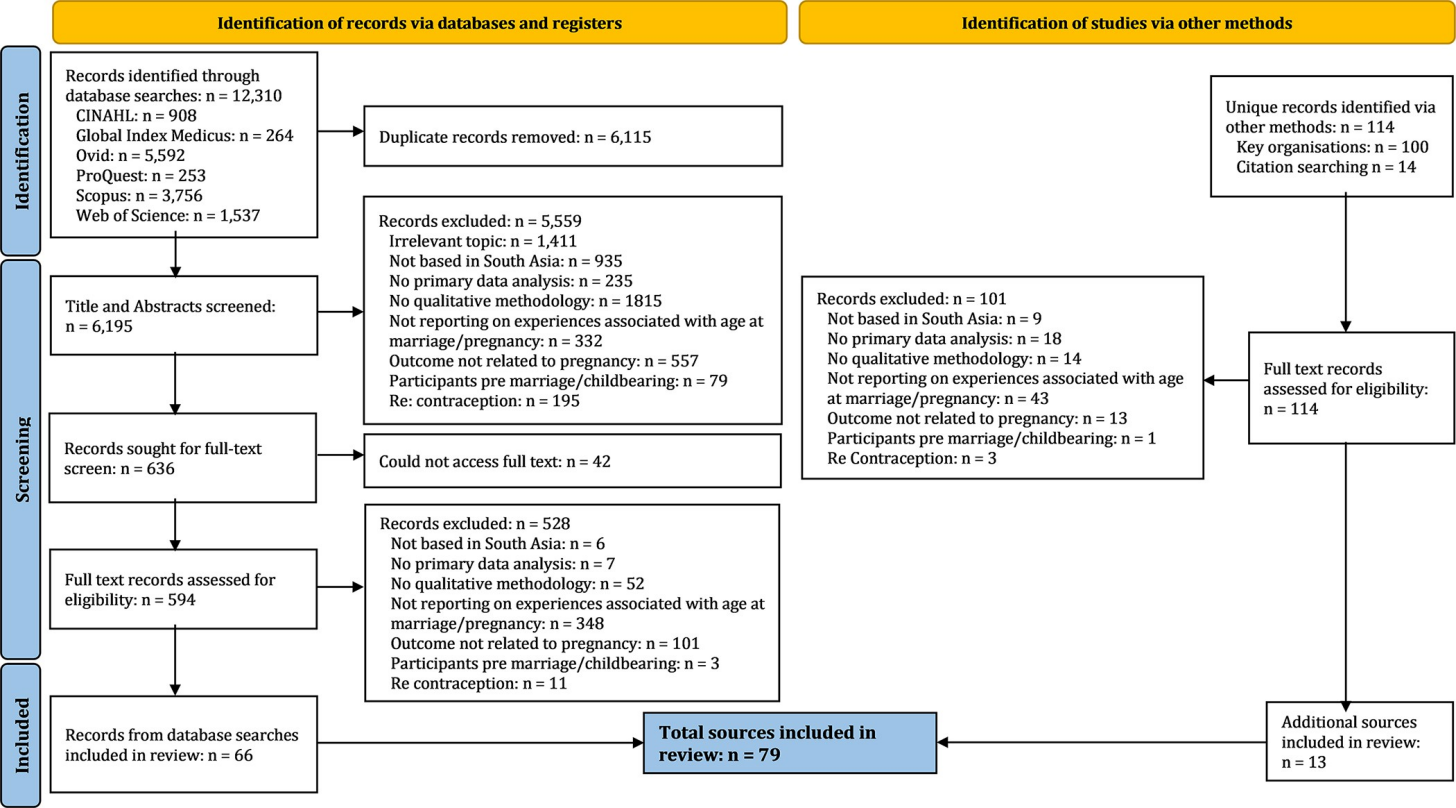
PRISMA flow diagram presenting screening for the review [123].

**Fig 2:**
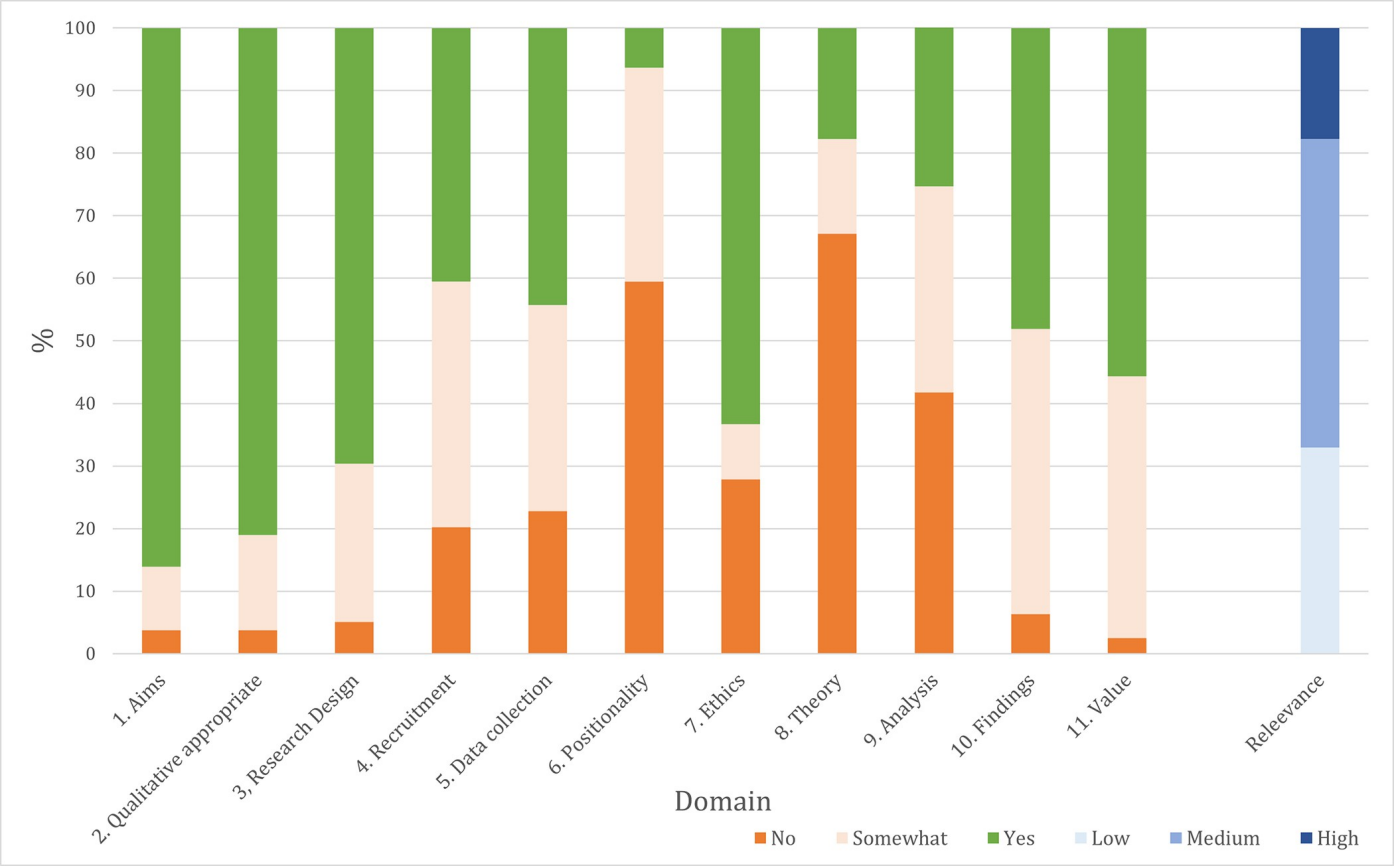
Summary of responses for each question from the Critical Appraisal Skills Programme (CASP) tool and overall relevance for all included studies (n = 79).

**Fig 3:**
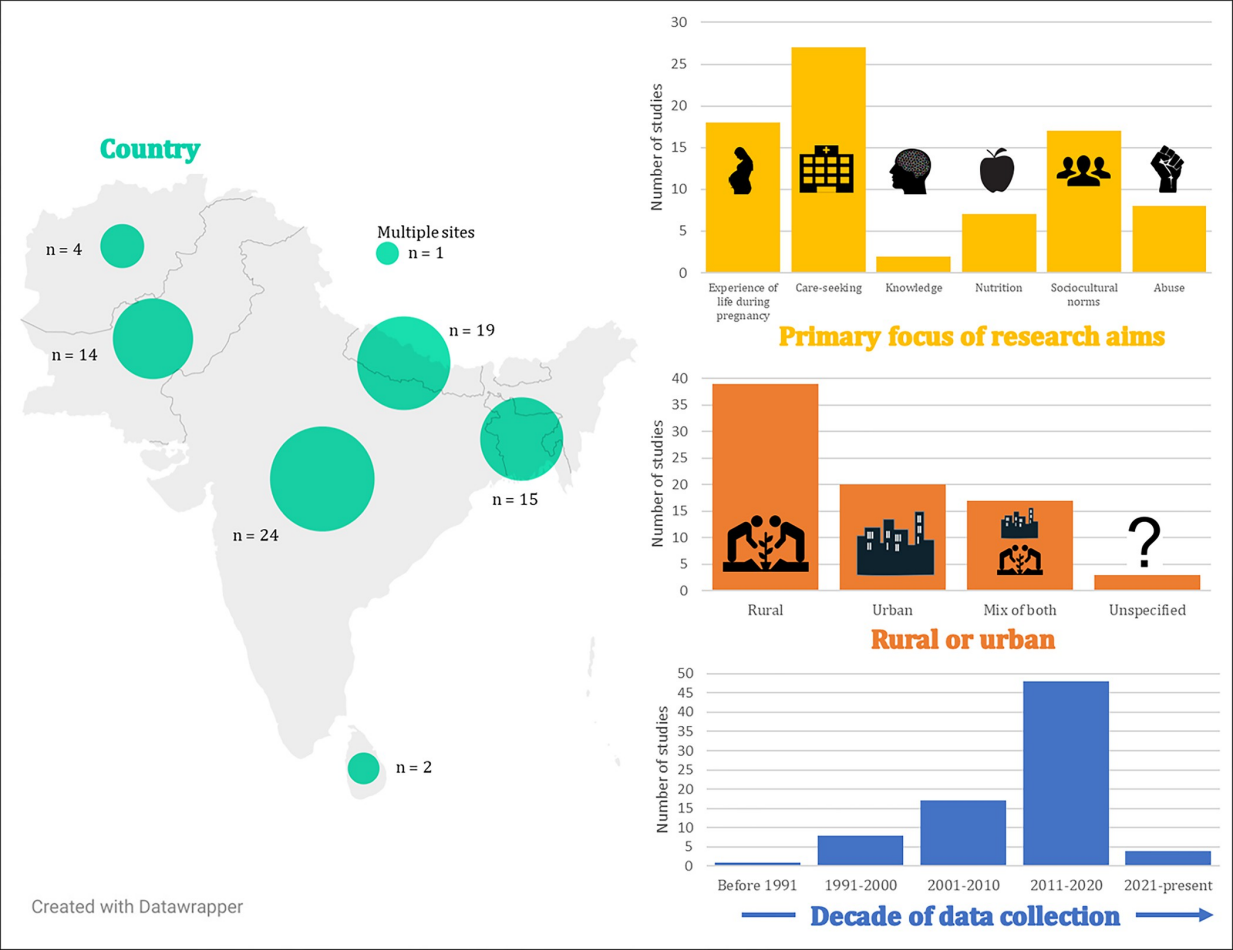
Summary of study characteristics of the included studies, including country, focus of research aims, location, and decade of data collection. The map was produced using datawrapper.de and the icons included in the graphs obtained from openclipart.org/. The authors hold the full copyright for these images.

Of the 6,195 papers identified through database searching, we included 66 (Fig 1). We identified an additional 13 grey literature sources, resulting in 79 sources in total. According to our judgement, >80% of sources adequately reported on their research aims and justified their qualitative approach, whereas <20% adequately reported on positionality and theoretical underpinnings (Fig 2 and S2 Appendix). We deemed 14 sources ‘high’ relevance [44–57], 39 ‘medium’ [58–96], and 26 ‘low’ [97–122]. 24 studies were conducted in India (10 from Southern/Central India, 10 from Northern India, 4 from multiple sites), 19 from Nepal (11 from Hills/Mountains, 7 from the Terai, 1 from multiple sites), 15 from Bangladesh, 14 from Pakistan, 4 from Afghanistan, 2 from Sri Lanka, and 1 from multiple countries (Fig 3). Notably, no high relevance studies were undertaken in India, whereas a high proportion were undertaken in Bangladesh (n = 6) and Nepal (n = 5). Research sites were in rural settings in 39 studies, urban in 20, and either a combination of both or unspecified in 20. Broadly, research aims focused on care-seeking (n = 27), experience of life during pregnancy (n = 18), sociocultural norms relating to maternal health (n = 17), abuse (n = 8), nutrition (n = 7), and knowledge (n = 2), however studies often reflected on a range of factors (Fig 3). The evidence base has increased with each decade, with two thirds of studies collecting data since 2010. Table 2 summarises the characteristics of high relevance studies (medium and low relevance studies presented in S3 Appendix).

**Table 2:** Summary of high relevance studies.

*Author*	*Title*	*Country (Region)*	*Participants*	*Methods*	*Outcome/area of focus*
Rajbanshi et al. (2021)	Perceptions of good-quality antenatal care and birthing services among postpartum women in Nepal	Nepal (Morang)	14 adolescents with a high-risk pregnancy who did not comply with hospital referrals: pregnant or within 42 days of birth	IDI *Thematic analysis*, *within behaviour change theory*	Reasons for non-adherence to hospital referral
Maharjan et al. (2019)	Factors influencing the use of reproductive health care services among married adolescent girls in Dang District, Nepal: A qualitative study	Nepal (Dang)	IDI: 14 Adolescent girls unknown # FGD (6-8/group) 10 KII	IDI, KII, FGD *Systematic text condensation*, *no theoretical framework presented*	Knowledge of health care-seeking practices among married adolescent girls
Paudel et al. (2018)	A qualitative study about the gendered experiences of motherhood and perinatal mortality in mountain villages of Nepal: Implications for improving perinatal survival	Nepal (Mugu)	63 IDI: 42 women and families, 10 nurses/auxiliary nurses, 2 FCHV, 2 support staff, 1 auxiliary health worker, 2 traditional healers, 4 stakeholders (2 journalists and 2 activists)	IDI *Thematic analysis*, *sociocultural framework*	Experiences and beliefs of perinatal death
Shahabuddin et al. (2019)	Maternal health care-seeking behaviour of married adolescent girls: A prospective qualitative study in Banke District, Nepal	Nepal (Banke)	52 IDI: 22 married pregnant adolescents, 10 married non-pregnant adolescents, 7 FCHV, 1 Government health officer, 1 health post worker, 1 medical doctor at hospital	IDI and FGD *Socioecological framework guided analysis*	Adolescent use of maternal health services
Cameron (1998)	On the edge of the auspicious: The practice and meaning of gender and caste in rural Nepal’s low-caste households and society	Nepal (Bajhang)	Unknown # life histories: Women of different castes, including low-caste specialists	IDI for life histories, ethnographic observations, and engagement *Narrative analysis supplemented with cultural texts*, *theoretical framework centring caste and gender*	How gender and caste intersect in social experiences
Pike et al. (2021)	Family influences on health and nutrition practices of pregnant adolescents in Bangladesh	Bangladesh (urban slum in Dhaka and rural Rangpur)	192 IDI: 96 pregnant adolescents or with <1 y/o, 64 family members, 32 healthcare providers	IDI *Identifying themes and gaps within socioecological framework*	Experiences of pregnant adolescents (ANC, nutrition, role of family)
Samandari et al. (2020)	Understanding individual, family and community perspectives on delaying early birth among adolescent girls: Findings from a formative evaluation in rural Bangladesh	Bangladesh (Kurigram Sadar and Rajarhat, Kurigram)	20 unmarried girls <20yr, 21 newly married girls <20yr (within 1yr), 14 husbands, 47 influential adults, 15 community leaders, 10 community health providers	IDI *Thematic analysis*, *relating to theory of change*	Social norms and barriers/facilitators to delaying first birth
Shahabuddin et al. (2017)	Exploring maternal health care-seeking behaviour of married adolescent girls in Bangladesh: A social-ecological approach	Bangladesh (Rangpur)	IDI: 25 married pregnant adolescents, 10 married non-pregnant adolescents 3 FGD: 1 with 6 CHW, 1 with 7 community members, 1 with 6 MIL 4KII: 1 government officer, 2 NGO officials, 1 doctor	IDI, FGD and KII *Socioecological framework guided analysis*	Married adolescents’ knowledge, perception, and use of reproductive and maternal health services
Rashid (2011)	Human rights and reproductive health: Political realities and pragmatic choices for married adolescent women living in urban slums, Bangladesh	Bangladesh (slum in Dhaka)	>60 IDI: 50 married adolescent girls, 12 husbands, additional family members 8 case studies: married adolescent girls	Ethnographic interviews, observations and interactions *Phenomenological approach*	How slum life impacts women’s reproductive behaviour
Rashid (2006)	Emerging Changes in Reproductive Behaviour among Married Adolescent Girls in an Urban Slum in Dhaka, Bangladesh	Bangladesh (slum of Dhaka)	>60 IDI: 50 married adolescent girls, 12 husbands, additional family members 8 case studies: married adolescent girls 19 IDI: 6 slum leaders, 8 NGO workers, 1 clinic paramedic, 1 religious leader, 3 health workers	Ethnographic interviews, observations and interactions *Phenomenological approach*	Life histories and reproductive health histories
Schuler et al. (2006)	The timing of marriage and childbearing among rural families in Bangladesh: Choosing between competing risks	Bangladesh (villages in Rangpur and Magura)	85 IDI: 20 men, 65 girls/women 24 FGD (unknown #/group): 4 groups	IDI and FGD *Grounded theory*	Norms, practices and decision-making related to marriage and pregnancy timing
Kamran et al. (2019)	Situation Analysis of Reproductive Health of Adolescents and Youth in Pakistan	Pakistan (Peri-urban areas in Islamabad, Lahore, Karachi, Peshawar, and Quetta)	> 250 adolescent boys and girls: 40 IDI, 24 FGD (~9/group) total unknown #	FGD and IDI *Grounded theory*	Cultural norms around reproductive health of adolescents
Hamid et al. (2009)	Who am I? Where am I? Experiences of married young women in a slum in Islamabad, Pakistan	Pakistan (urban slums, Islamabad)	10 IDI: married adolescent women (conducted over multiple visits)	Unstructured interviews, married life calendar Narrative structuring, *Bronfenbrenner’s ecological framework*	Knowledge of married life and reproduction at time of marriage
Perera et al. (2018)	’When helpers hurt’: Women’s and midwives’ stories of obstetric violence in state health institutions, Colombo district, Sri Lanka	Sri Lanka (Colombo)	5 FGD: 28 PHM 6 FGD: 38 pregnant women (6-7/group)	FGD *Data organised within intersectionality framework*	Intersection of age, social class, and cultural background with obstetric violence

Abbreviations: ANC; antenatal care, CHW; community health worker(s), FCHV; female community health volunteer(s), FGD; focus group discussion(s), IDI; in-depth interview(s), KII; key-informant interview(s), MIL; mother(s)-in-law, NGO; non-governmental organisation; PHM; public health midwife(s), y/o; year old, yr; year(s), unknown #; unknown number.

### Themes

**Fig 4:**
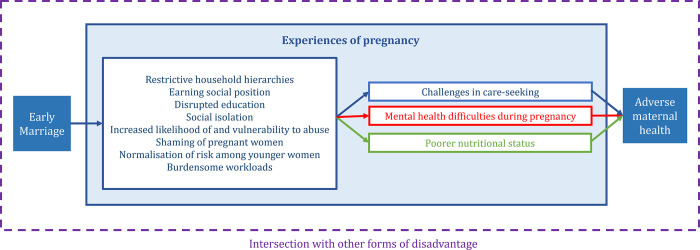
Representation of the themes through which early marriage and early pregnancy influences the care-seeking, nutrition, and mental health of women and girls during pregnancy.

We grouped experiences of pregnancy into three categories: care-seeking, mental health, and nutrition (Fig 4). We identified eight themes through which early marriage and early pregnancy affect experiences, relating to: restrictive household hierarchies, earning social position, knowledge of pregnancy needs, social isolation, increased likelihood of and vulnerability to abuse, shaming of pregnant women, normalisation of risk among younger women, and burdensome workloads (S4 Appendix). We narratively describe each theme, the evidence on how early marriage and/or early pregnancy affects each theme, and how the theme affects each category.

#### Early marriage intersecting with other identities

Within each of the following themes, persistent sociocultural hierarchies, such as socioeconomic status and caste group, shaped the pregnancy experiences of women and girls, moderating outcomes following early marriage and early pregnancy.

Women and girls from more disadvantaged caste or ethnic groups faced persistent challenges relating to their health and well-being. They were more likely to experience discrimination from healthcare providers, moderating experiences of abuse following early marriage [44, 46, 63, 71, 107, 113, 122]. However, Mary Cameron found that women and girls from more disadvantaged caste groups in rural Nepal were more involved in choosing a spouse than those from advantaged caste groups, improving marital relationships which were otherwise negatively affected by early marriage [44]. Women from advantaged caste groups also faced tighter restrictions to their movement and behaviour during pregnancy, compounding with the effect of early marriage [44, 91].

Poverty was commonly cited as a driver of early marriage and mediator of experiences. Financial stressors, such as unpaid dowries and debt from loans, increased anxiety during pregnancy for those married early [46, 49, 52, 53, 56, 67, 71, 74, 83, 84, 87, 90, 113]. Many feared they would not be able to seek care due to financial constraints [44, 46, 49, 52, 53, 56, 67, 71, 84, 91], as expressed by this pregnant adolescent:

*“I wanted to call the doctor*. *I was so sad that my husband said we should wait longer*. *I was trying so hard*. *I didn’t want to go through so much pain just so we wouldn’t have to spend money*.” Rural Bangladesh, 2009 [84]

Financial vulnerabilities made pregnant women and girls feel more dependent on their family, exacerbating restrictive hierarchies among those married early [44, 45, 52, 53, 63, 65]. Financial vulnerabilities also limited the options available to women and girls wanting to escape an abusive marriage, which was more common among those married early [52, 53, 63, 65, 83].

However, families often gave financial reasons for not accessing care, despite services being provided for free or there being financial incentives available [44, 49, 56, 85, 90], as reported by this author reflecting on a girl whose father-in-law refused treatment despite offers of free transport:

*“The father-in-law was responsible for the final decision*, *and he decided against it*, *saying that “We do not have the money to cover the expenses”*, *even after the local men had offered to carry her there for free… the baby finally came out*, *stillborn*, *(and the mother) died*.” Rural Nepal, 1988/89 [44]

#### Family hierarchy preventing women and girls from speaking up and being listened to

The most common theme we identified in this review related to the ways in which social hierarchies, which persist within households across South Asia, limit the ability of women and girls to make decisions during pregnancy. Young pregnant women and girls felt unable to make decisions in several domains during pregnancy, including what they ate, what work they undertook, who they interacted with, when to seek care, and which care they received [45, 46, 49, 56, 73, 75, 81, 83, 96]. Instead, decisions were made collectively by the household, with their mother-in-law generally having the final say in reproductive matters [44, 47, 49, 56, 57, 73, 85, 107]. Most commonly, their husbands were not very involved in decision-making relating to reproductive health due to norms preventing their involvement [44, 47, 51, 52, 61, 63, 75, 115], as explained by this author of a study in rural Pakistan:

“*In joint families… a man is considered besharam (shameless) if he exhibits an ‘excessive’ interest in his pregnant wife”* Author, 2001 [75]

Women of all ages had their decision-making ability restricted by household hierarchies; however, restrictions were stricter for younger women and girls and those who were married early. Families considered younger girls less mature and those married early less knowledgeable [46, 49, 51, 52, 59, 77, 112, 113], justifying their increased control over decision-making, as expressed by this 26-year-old aunt:

“*You are of a young age*, *whereas we are elder and we know more”* Urban Bangladesh, 2019 [49]

*Impact on care-seeking*. In most regions, young pregnant women and girls were often excluded from decision-making about care-seeking during pregnancy as this was their mother-in-law’s domain [46, 47, 56, 57, 104, 113], as expressed by this adolescent mother discussing their decision to attend ANC services during pregnancy:

“*I have to stay quiet*… *I can’t say anything*, *they are senior to me”* Nepal, 2006 [96]

Care-seeking preferences of household-heads were often prioritised, even when these conflicted with healthcare provider advice [44, 49, 56, 85]. In extreme cases, women were not listened to in critical situations, resulting in complications which may have been preventable [44, 47, 80, 84], as was the case with this 17-year-old mother:

“*I knew that my condition was very serious*, *and everyone kept on telling me to try having the baby at home*. *I was trying*, *and I knew I couldn’t try anymore*, *but the others didn’t understand how serious it was*.*”* Rural Bangladesh, 2009 [84]

We identified two ways in which household hierarchies affected care-seeking among younger women and girls and those married early: i) younger women and girls felt less able to share their concerns and participate in care-seeking decision-making due to their lack of agency or shyness [52, 59, 62, 75, 80, 85, 93, 96, 113], and ii) household members were less likely to listen to their care-seeking requests due to a disbelief or distrust in younger daughters-in-law [44, 47, 49, 54, 56, 57, 73, 84].

However, there were regional differences. For example, the mothers-in-law of pregnant women and girls in Central and Southern India and Sri Lanka appeared less likely to enforce their beliefs [104, 117], as expressed by this mother-in-law from a study undertaken in South India:

“*Now*, *I don’t stop my kids from going to the hospital for delivery but I think it is unnecessary”* 2021 [117]

*Impact on mental health*. Having their decision-making restricted during pregnancy was a major source of stress for young pregnant women and girls and those married early, as any conflicts with household heads prevented them from acting in line with their interests [51, 61, 63, 70, 84, 91, 93, 113]. Women were frustrated at being perceived as uneducated by household members who excluded them from decision-making and by healthcare providers who treated them with disrespect [49, 51, 93].

Pregnant women and girls were fearful of experiencing complications during pregnancy and felt helpless due to their lack of control [60, 84, 85, 91]. This is highlighted by this young woman reflecting on her experience of delivery:

“*My husband and his mother did not agree with the recommendations of ‘dai’ to take me to the hospital*… *I was pretty afraid*, *but I had to stay quiet due to the pressure of my husband and his family”* Rural Pakistan, 2013 [85]

Women and girls reported that navigating hierarchies in their new household during adolescence and while pregnant was overwhelming [51, 58, 83], such as this girl who married at age 12:

“*I was married off at a very early age*, *brought into a new family*, *I had to adjust with a new family and with my husband*. *Everything was haphazard and in the middle of all those changes I became a mother too*. *It was all very overwhelming”* Rural Bangladesh, 2013–14 [58]

*Impact on nutrition*. Young pregnant women and girls often relied on other household members to satisfy their nutritional needs; household members advised them what to eat, bought them food or provided them with money to buy food, decided on the allocation of food between family members, and influenced when they were able to rest during pregnancy [49, 60, 64, 71, 91]. Food allocation was negatively affected by norms around women eating last and sacrificing their share for their family [49, 64, 91], as was the case with this woman with a young child:

“*Who will cook again*? *My stomach is full if my family’s stomach is full*. *I don’t get hungry”* Nepal Terai, 2019 [91]

#### Girls being pressured into pregnancy to earn social position within their household

Women and girls felt pressure to increase their social position through marriage and delivering a baby, preferably a son [45–47, 52–54, 63, 70, 90, 94, 108]. These norms drove early marriage and repeated pregnancies, as explained by this woman who lost her baby at age 15 when asked why she got pregnant repeatedly at such a young age:

“*I know it is not good for my health*. *But my parents-in-law said to me that I should produce at least one child*. *Otherwise*, *they would bring a co-wife to my husband*.*”* Rural Nepal, 1998 [90]

Women and girls with poor marital relationships, common among those married early, felt these pressures more strongly as having a child was often viewed as a way to strengthen relationships [47, 52, 53, 55, 90, 93, 119]. Young and newly-married daughters-in-law were generally less trusted by their marital family, intensifying these pressures [45, 46, 52, 55, 85, 93, 108], as explained by this case study of a woman married at age 14:

“*My mother-in-law says I am not innocent and my husband says that I have to learn to be obedient and submissive”* Urban Pakistan [45]

*Impact on care-seeking*. The needs of young women and girls who had not yet ‘earned’ their social position were often not taken seriously by household members, particularly during their first pregnancy [52, 54]. Family members often did not trust women and girls when they had health complaints [47, 53, 63, 83], as reported by this teacher:

“*When she tells about her illness*, *the mother-in-law often does not trust; mother-in-law thinks that she is telling a lie (Nauragareko)”* Rural Nepal, 2015 [47]

*Impact on mental health*. The pressures felt by young girls increased their anxiety [44, 45, 47, 70, 73, 74, 94, 108], as explained by this author reflecting on the mental health of early married women in Northern India:

‘*Such experiences were also recounted by other women and that it had caused them sadness and anxiety due to the immense social and familial pressure on them to prove their fertility and produce children soon after marriage*.*’ 2015–7* [94]

Women and girls were commonly blamed if they could not conceive or had experienced miscarriages, and families often provided threats such as divorce or a co-wife [46, 47, 52, 53, 55, 90, 93, 107, 119]. Adjusting to and living with these pressures was particularly upsetting for younger women and girls, who were grappling with establishing their identity during adolescence [45, 83].

*Impact on nutrition*. Some pregnant women and girls saw fasting as a way of pleasing their in-laws and improving their chances of having a son [108].

#### Early marriage affecting knowledge of pregnancy needs

Girls usually left school before or shortly after marriage, negatively affecting their knowledge of reproductive and maternal health which was often taught in school [45, 46, 48, 49, 51, 54–56, 58, 60, 69, 80, 100, 109, 113, 115]. This 17-year-old mother expressed how her lack of education negatively affected pregnancy preparedness:

“*Who would do it if one knew everything*? *If I had known everything*, *I would not be pregnant”* Rural Nepal, 2015 [46]

However, even when women and girls were educated and understood their needs during pregnancy, they were often not able to raise their needs to household decision-makers, due to shyness, lack of agency, feeling overwhelmed, and/or thinking that they would not be listened to [46, 57, 70, 83, 93, 120]. Often, even girls who spoke out were not listened to [70, 73, 97, 108], such as this girl from a study in rural Uttar Pradesh who had married at age 13:

“*I understand the importance of doctors*. *I wanted to deliver my first child in the hospital*, *but my mother-in-law did not allow me to go”* Rural India [73]

*Impact on care-seeking*. Younger and less educated women were often less aware of warning-signs during pregnancy [17, 46, 49, 50, 55, 58, 80, 92, 106, 109]. Some women did not realise they were in labour as they did not recognise their labour pains and relied too heavily on their delivery date (which are known to be inaccurate due to recall error) [51, 58, 62, 69, 80, 115], such as this woman who experienced a neonatal death during adolescence:

“*At home*, *I was in labour pain for three days*. *It contracts and leaves*. *I thought the date had not yet approached*. *That’s why I didn’t even tell my husband*.*”* Nepal, 2019–2020 [51]

When accessing ANC services, uneducated women were often scolded by providers for their lack of knowledge, negatively affecting experiences and future care-seeking as a result [46, 49, 56, 80].

Households often prioritised the opinions of older female relatives over the perspectives of pregnant women, even when pregnant women were educated or their relatives’ opinions conflicted with healthcare provider advice [49–51, 56, 69, 76, 85, 93, 97]. Healthcare providers considered this a barrier to the care they provide [49, 56, 113], as expressed by this nurse:

“*Family members are superstitious and tell*, *‘this is nonsense what the doctor says*. *Follow what we say*.*’”* Urban Bangladesh, 2014–15 [56]

*Impact on mental health*. Young women and girls and those married early acknowledged their lack of knowledge and expressed a sense of helplessness [49, 58, 60, 65, 88, 93, 107], such as this 15-year-old pregnant girl:

“T*hose who are underage… we understand less*, *or*, *we do not have the ability to understand”* Rural Bangladesh, 2019 [49]

Furthermore, large education gaps were common among couples who married early, which negatively impacted spousal communication and therefore their mental health during pregnancy [46, 58, 60, 108, 112].

*Impact on nutrition*. Less educated girls were less informed of their increased nutritional requirements during pregnancy [49, 50, 64, 108], allowing for misinformation to be perpetuated regarding food to be avoided and the benefits of physical labour during pregnancy [49, 50, 68, 90].

#### Girls feeling isolated during pregnancy

Married women and girls commonly faced restrictions to their social interactions and independence. Studies from Northern India, Nepal Terai, Pakistan, and Bangladesh reported that girls were often prohibited from leaving the house unaccompanied [54, 74, 76, 81, 85, 91, 96]. This was not reported by girls in Southern India, although they still faced restrictions on their social interactions and behaviours [82, 93]. These restrictions increased during pregnancy due to the associated shame and stigma [49, 54, 59, 81, 82, 91, 104].

While most women experienced some level of restriction on their social behaviour, these were often tighter for younger women who were considered more vulnerable to influence [45, 51, 54, 70, 76, 82, 88, 91, 112], as expressed by this married adolescent reflecting on her path to early motherhood:

“*I do not go anywhere*, *even places near my house*, *because my mother-in-law suspects that if I talk to anybody*, *I might be badly influenced by them*.*”* Rural Bangladesh, 2017 [54]

Younger women found the navigation of relationships within their marital household difficult due to their shyness [45, 51, 76, 80, 87, 112, 120]. Furthermore, women who married early were often much younger and less educated than their husbands, negatively affecting how accepted they felt in their marital household [46, 58, 60, 108, 112].

Women and girls who had a close relationship with their natal family coped better with tensions within their marital household, partly because natal family relationships affected spouse choice but also because their natal household provided a place to visit during times of stress or in cases of abuse [45, 54, 63, 65, 104], as for this 19-year-old pregnant girl in an abusive relationship:

“*So*, *whenever we have a big argument I visit my maternal home”* Urban Nepal, 2013 [63]

*Impact on care-seeking*. In studies from Northern India, Nepal Terai, Pakistan, and Bangladesh, women were often not permitted to leave home unaccompanied, and therefore relied on their relatives to attend healthcare appointments with them [54, 74, 76, 81, 85, 91, 96]. If their relatives were unsupportive of care-seeking, this negatively impacted their ability to seek care [61, 93, 96, 120], as with this pregnant adolescent:

“*ANC check-up is not easy for me*. *I have problems at home*. *[My in-laws] won’t allow me to go outside much*. *I cannot go for ANC check-up on my wishes*. *They said that I do not need to go again*.*”* Nepal, 2006 [96]

However, some participants were allowed to visit facilities with neighbours or relatives of a similar age, overcoming some of the restrictions to unaccompanied mobility [49, 56, 57, 87, 97], as this teenage mother who lived far from the ANC centre reflected on:

“*I had a pregnant neighbour with close delivery dates*. *We would go together”* Rural Nepal, 2014–15 [57]

*Impact on mental health*. Transitioning directly from relative freedom during childhood to restrictions in marriage was difficult [49, 54, 70, 81, 87, 88, 112], as explained by this pregnant adolescent when explaining how pregnancy has impacted her daily life:

*“I was able to live independently*. *Now I walk less*, *hang around less; I have to sit at home*, *can’t go anywhere outside*. *Can’t live willingly*.*”* Urban Bangladesh, 2019 [49]

Young women and girls commonly reported feeling lonely and helpless during pregnancy due to a lack of social support [45, 47, 49, 51, 61, 70, 81, 88, 112], such as this 17-year-old woman who was continuing an unwanted pregnancy against her wishes:

“*I am feeling lonely and helpless*… *I am so anxious about it*. *I am feeling sick*. *I have fear*. *I am worried*.*”* Rural Nepal, 2010 [70]

*Impact on nutrition*. Young women and girls were generally unable to travel to local markets to purchase nutritious food, needing to rely on other household members to do so [49, 91].

#### Increased threat of abuse for adolescents

Emotional, sexual, and physical abuse was normalised among married women and girls across South Asia [45–47, 52, 53, 63, 65, 67, 73, 78, 79, 81, 89, 94, 110], to the extent that this early-married woman living in an urban slum in Pakistan viewed sexual abuse as a symbol of love:

“*Unwanted sex is also a symbol of love*. *It is a way to resolve the dispute between husband and wife*.*”* 2013 [78]

Girls were commonly subjected to abuse from multiple household members, increasing their vulnerability [45–47, 65, 89], as in this case study of an early-married woman:

“*He complained to his mother who told him to tie me up and have sexual activity with me anyway*.*”* Urban Pakistan [45]

For some, abuse intensified during pregnancy [52, 63, 65, 78, 79, 107] and injuries sustained from violent physical and sexual acts were suspected to have caused women and girls to miscarry [67, 78, 79, 81, 89, 110], as was the case for this early-married pregnant woman:

“*Even last night they beat me*, *and I am bleeding*. *I am four months pregnant and I am bleeding… he uses his hands and he throws me here and there against the walls”* Nepal [67]

Younger and early married women were more likely to be subjected to abuse [48, 65, 67, 73, 79, 81, 83, 89, 94, 110]. Having a much older or more educated husband, common among early married women, intensified abuse due to poor spousal communication [65, 73]. Girls in love marriages were also more likely to be subjected to abuse, as their in-laws were less likely to be accepting of their marriage [52, 53, 63]. Younger girls were also considered less physically and emotionally strong, and therefore less able to endure abuse [55, 73, 89].

*Impact on care-seeking*. Women and girls who were subjected to abuse may have been less likely to seek care; some delayed telling their household members about their pregnancy due to fear of retaliatory abuse [73], while the families of others prevented care-seeking out of fear that they would speak out [89], as reported by the author of this study in Karachi, Pakistan:

“*Her husband agreed to hospitalization*, *but he threatened her with divorce if she divulged any information about his violent behaviour to the hospital staff”* [89]

Girls also frequently experienced verbal abuse and neglect from healthcare providers, affecting the quality of care provided and future care-seeking [45, 46, 48, 49, 53, 57, 69, 71, 89, 95, 101, 113]. They were scolded by providers for marrying early, becoming pregnant early, having repeated pregnancies, and being less likely to follow their advice [46, 48, 49, 52, 53, 57, 62, 69, 95, 113]. Women and girls felt helpless in these situations, as expressed by this adolescent mother:

“*I was left alone in the stirrups legs up*. *I was screaming and nobody came*.*”* Urban Afghanistan, 1996 [69]

*Impact on mental health*. Being abused made women and girls feel scared and unsafe, negatively impacting their mental health [45, 52, 53, 63, 65, 79, 81, 89, 94], as expressed by this 17-year-old pregnant girl:

“*He beats me till he cools down and blames my maternal home for giving birth to me*. *I feel so sad and I regret marrying him (tears in her eyes)” Urban Nepal*, *2013* [63]

Younger women were more emotionally vulnerable to these effects, as reported by this author reflecting on healthcare-provider violence:

“*Young women having their first babies were particularly distressed but their cries for help*, *for their mothers or for God were largely ignored*.*”* Urban Afghanistan, 2010–12 [101]

Women and girls who were not able to return to their natal household suffered in particular, as this was a common fall-back option [46, 47, 63, 65, 89, 94, 110].

*Impact on nutrition*. Women and girls often felt sick and did not feel like eating due to stress caused by the abuse [63, 70]. Some women had their food withheld by abusers, severely impacting their nutritional status [52, 73, 89], such as this woman who married at age 13 years:

“*I was young and weak*, *I opposed him*, *he used to beat me*, *stopped giving me food*, *even tortured me during my periods… I wish I was never born”* Rural India [73]

Furthermore, women and girls were commonly threatened with abuse if they did not work hard enough, causing them to become overworked and exhausted during pregnancy [44–46, 59, 94].

#### Pregnancy being a time of shame

Across South Asia, women and girls felt conflicted as they transitioned from their unmarried state, when their bodies represented modesty and chastity, to pregnancy, when their bodies represented sexual activity [44, 56, 57, 72–74, 76, 83, 89, 90, 99, 104]. Studies from Pakistan, Bangladesh, Northern India and Nepal reported that pregnant women and girls often had their movements restricted by their families as a result [56, 57, 73, 76, 85]. This author explained how in Pakistan, a pregnant belly represents sexual activity:

“*Pregnancy*, *an obvious manifestation of sexual activity*, *is associated with ‘sharam’ [shame]*. *Pregnant women should avoid public space”* Rural Pakistan, 2001 [76]

Related to this, pregnant women and girls generally felt embarrassed and ashamed when discussing their pregnancy [17, 44, 72, 83, 89, 91, 99, 104, 115]. Women who were younger, less educated, and had a faster transition from childhood to pregnancy tended to be more shy and embarrassed when discussing their pregnancy [44, 46, 62, 72, 83, 91, 99, 120].

*Impact on care-seeking*. Women and girls frequently felt uncomfortable sharing pregnancy-related health concerns with their in-laws, meaning they delayed care-seeking [57, 89, 91, 104, 120], as with this young daughter-in-law living in rural Maharashtra:

“*I had red discharge and pain in my abdomen*. *I was ashamed of telling (my mother-in-law)*.*”* 1996 [103]

Pregnant women and girls often felt ashamed to seek care [46, 56, 57, 62, 73, 90, 96], particularly from male doctors, as reported by this pregnant girl aged 15:

“*I will be ashamed*. *The doctors see your body*. *A lot of people see your body*. *That’s why I didn’t feel like going to the medical*.*”* Rural Bangladesh, 2014 [56]

*Impact on mental health*. Women and girls found it difficult to manage their conflicting and shameful feelings during pregnancy, particularly as they felt unable to share their concerns with others [45, 58, 94, 104, 108, 113], as represented by this case study of an early married woman in Pakistan:

“*I am not innocent and my husband says that I have to learn to be obedient and submissive*. *I am not allowed to leave home unaccompanied*. *I worry for myself*. *Where is my home and who am I*?*”* [45]

#### Risk being normalised among younger pregnant women

Across South Asia, younger girls were thought to have an increased risk of experiencing complications during pregnancy and delivery due to their physical immaturity [21, 48–50, 54, 59, 60, 65, 68, 71, 74, 85, 88, 95, 98, 100, 102, 105, 109, 116, 119, 121]. This was attributed to early marriage [47, 51, 55, 66, 77, 88, 110].

Therefore, younger women experienced pregnancy with an expectation and normalisation of risk based on the experiences of friends and relatives, while feeling powerless to act to improve their outcomes [44, 47, 51, 60, 73]. This expectation is expressed by this young pregnant woman in rural Nepal:

“*Well*, *I think I am going to die anyway*, *so what does it matter if I have a son*? *All I wish is that it lives*. *Will it live or die*? *Nobody knows*.*”* 1988/89 [44]

*Impact on care-seeking*. Women and girls often delayed care seeking as a result of this normalisation of risk, as they considered pregnancy to be a condition needing care only in complicated cases [46, 47, 49, 56, 58, 62, 71, 74, 77, 80, 85], as explained by this young married woman:

“*As I had a normal delivery*, *I never visited the doctor because there were no complications*.*”* Urban India, 2020–21 [71]

Many considered younger women to have an increased risk of needing a caesarean-section, particularly in Bangladesh [49, 55, 56, 60]. This pregnant adolescent explained that she planned to deliver at home for this reason:

“*There are a lot of problems if you go to the hospital*, *if you do a C-section there is a problem in moving*. *There is a problem in eating*, *for three to four months you can’t do any heavy work”* Rural Bangladesh, 2014–15 [56]

*Impact on mental health*. Stories of adverse outcomes among friends and relatives were upsetting for young girls [44, 60, 70, 71, 73, 81, 88]. Coupled with the fact that they were generally unable to make decisions to improve outcomes, women and girls often felt anxious about this normalisation of risk [50, 60, 70, 71], as with this young mother whose friend had died during delivery:

“*I was very afraid during delivery because I thought I would die… One of my friend expired (died) during delivery at the age of 16…”* Urban Nepal, 2015 [60]

#### Conflicting reproductive, domestic, and economic roles

Many women and girls faced demanding domestic workloads within their marital households, often with additional responsibilities from agricultural and income generation work [44–47, 49, 52, 55, 59, 64, 68, 80, 83, 90, 94, 108, 110, 112, 120]. The extent to which their health and wellbeing during pregnancy was affected depended on the type of work and the extent to which their workload expectations aligned with their household’s expectations [44, 46, 47, 49, 52, 53, 55, 58, 110].

In rural environments, women and girls were often engaged in agricultural work, which families generally expected to continue throughout pregnancy [44, 46, 55, 59, 68, 83, 90]. This work was often dangerous, and accidents could cause miscarriages or stillbirths [44, 46, 47], as this lady health worker described:

“*She had gone to bring fodder from the* forest. *She slipped on the road*, *hurt her abdomen*. *She had bleeding and fainted… Fortunately*, *the mother survived; however*, *the baby died”* Rural Nepal, 2015 [47]

In urban environments, women were often involved in income generating work [52, 53, 55, 58]. Pregnancy was often delayed to prioritise income generation, or women were expected to give up work to start a family [52, 53, 55, 71, 110]. The expectation depended on the preferences of their in-laws, as expressed by this girl who married at age 14:

*“(My husband) really wanted a baby*. *I was working in the garments*. *I wanted to work for longer*, *save up some money and then have a child*… *My mother convinced me to have a baby to make him happy*.*”* Urban Bangladesh, 2001–03 [52]

Younger women and girls and those married early felt less able to negotiate with their in-laws regarding their workload [45–47, 49, 52, 53, 58, 64, 80, 90, 94, 112]. Younger mothers were also considered more vulnerable to exhaustion due to their physical immaturity [47, 50, 59, 71, 94, 108]. The families of women and girls who married early were also less likely to have invested in their education, limiting their employment prospects [45, 55, 58, 83].

*Impact on care-seeking*. Heavy workloads prevented women from seeking care [46, 47, 56], as was the case with this early married woman who delivered at home due to workload demands:

“*‘I also delivered the baby at home*. *I was working all morning and in the evening… Nobody was aware at home that I was having the labour pain*.*”* Rural Nepal, 2015 [46]

*Impact on mental health*. Women and girls were anxious due to their demanding workloads, and for the potential that pregnancy complications may prevent them from working after childbirth [47, 52, 58, 64, 94, 112]. Heavy workloads also prevented them from visiting their natal family [80, 110].

However, work outside the home also provided autonomy, freedom of movement, a social network outside the household, and some economic independence [52, 53, 55, 58, 75].

*Impact on nutrition*. Pregnant women and their families acknowledged the risks associated with demanding physical labour during pregnancy, such as exhaustion and anaemia [46, 47, 71, 90]. However, some believed that heavy physical labour was beneficial during pregnancy [68, 90], as expressed by this husband whose wife had recently delivered:

“Pregnant women do every kind of work. The harder your work during pregnancy, the easier it will be for delivery” Husband, rural Nepal [90]

## Discussion

This review found that several interconnected factors shape experiences of pregnancy following early marriage in South Asia. The two most important factors we identified were the social position of women and girls within their household, and the quality of their relationships within and outside of their home. By presenting how these interpersonal factors connect with other factors, such as education, experiences of abuse, and burdensome workloads, we provide a new perspective which highlights the importance of households and communities listening to and trusting women and girls.

Additional themes we found in this review highlight how, due to their young age and lack of education, respectively, younger and early married women felt more shy and less empowered and were considered less knowledgeable. These were used as justifications for households maintaining control over decision-making. Early married women struggled with the restrictions placed on their social interactions, as this prevented them from forming and maintaining relationships and exacerbated the transition from childhood to married life. Younger women were considered to have an increased risk of pregnancy complications due to their physical immaturity, leading to an expectation of negative outcomes which increased anxiety and negatively affected care-seeking behaviours.

Our findings should be considered within the limitations of this review. While we searched for studies from across South Asia, most studies were from India, Nepal, Bangladesh, and Pakistan, with few from Sri Lanka or Afghanistan, and none from Bhutan or the Maldives. The lack of perspectives from these regions limited our ability to draw comparisons on experiences of pregnancy between regions. Furthermore, findings may not apply to all regions within South Asia due to sociocultural and demographic differences between countries and sub-national regions. Furthermore, context-specific evidence may have been decontextualised while synthesising findings from studies across South Asia. As we aimed to identify potential pathways through which early marriage impacts maternal health, we may have had a negative bias when synthesising findings. However, we sought out contradictory evidence when developing themes to challenge this bias. Furthermore, by excluding studies exploring the experiences of married women and girls outside of pregnancy, we exclude the potential to explore relevant experiences before pregnancy. However, inclusion of these studies would have made the scope of this review too broad. We were limited in the extent to which we could disentangle experiences of early marriage and early pregnancy, as early marriage was considered a pre-requisite to early pregnancy and therefore, they were often considered together. Despite these limitations, this review provides new perspectives on the experiences of pregnancy following early marriage in South Asia. Of the three pathways we identified, the evidence base exploring care-seeking experiences was strongest, whereas qualitative evidence exploring experiences of mental health and nutrition was lacking.

Quantitative evidence from South Asia supports our finding that younger women are less involved than older women in decision-making during pregnancy [98, 124–127]. In this review, some girls reported actively diminishing their agency during household decision-making, suggesting they acknowledge the benefits of acting in line with rather than challenging hierarchies in decision-making within households [49, 85, 90]. This supports other conceptualisations of household bargaining in South Asia which highlight that an absence of protest does not reflect an absence of questioning [13, 128]. As household hierarchies frequently delayed or prevented care-seeking, we join others who call for an update to existing models which seek to understand care-seeking to incorporate the nuance of women’s decision-making capabilities [129, 130]. We found that newly married women and girls in particular had their decision-making restricted, as they have not yet gained status through their reproductive role. However, quantitative evidence from Nepal and Bangladesh suggests that the household status of women and girls does not increase with time or with the delivery of a child [131–134]. These nuances are context-specific and difficult to measure, requiring research at a local level, which we were unable to undertake in this regional review [135]. Programs to improve experiences during pregnancy in South Asia must address household dynamics, emphasising the importance of families listening to and trusting women and girls and prioritising their needs.

Current conceptualisations of experiences of pregnancy following early marriage focus on the importance of education [136–140]. This is supported by quantitative evidence from South Asia which has found associations between education, knowledge of reproductive health [19, 109–111], and decision-making [127, 131, 135, 141]. However, this review found that even educated girls were generally unable to speak up and be listened to within their households due to restrictive household hierarchies, meaning that education does not translate into improved experiences. This inconsistency may reflect the opposing mechanisms of caste and education; while women from disadvantaged caste groups are less educated, their households are described as more egalitarian meaning they may be more involved in decision-making [142, 143]. Efforts to improve the educational attainment of girls must be accompanied with efforts to shift household hierarchies and social norms for them to be able to enact learned knowledge.

Socioeconomic vulnerabilities are another commonly cited driver of early marriage and mediator of experience. Indeed, we found evidence to suggest that socioeconomic deprivation and unemployment limited the opportunities for women and girls outside of their marriage, making them less able to negotiate their needs within their household [52, 53, 71, 87]. Agarwal refers to this as a woman’s ‘fall-back position’, associating lower fall-back positions (fewer options if the marriage fails) with lower bargaining power within a household [128]. However, we also found that socioeconomic reasons were often given for not seeking care or following recommended nutritional practices, despite services and supplements being offered for free [44, 49, 56, 85, 90]. Inconsistencies within the quantitative literature on associations between socioeconomic status and pregnancy decision-making [131, 144, 145] may be explained by household dynamics, as sociodemographic factors are understood to be less important in contexts where women’s agency is more restricted [146]. These conflicting findings highlight the importance of understanding the local contexts in which women and girls live when undertaking research and designing programs.

Another set of interwoven factors relates to the societal shaming of sexual activity and the restrictions placed on the social interactions of women and girls. Our findings are consistent with the quantitative literature which supports an association between early marriage, social restrictions, and reduced care-seeking [146–148]. However, the extent to which these factors influenced pregnancy experiences differed between regions. Further efforts to harness the potential of social groups to deconstruct the shameful connotations associated with pregnancy should be explored to improve care-seeking and socioemotional wellbeing.

While we identified few qualitative studies focusing primarily on mental health during pregnancy, each theme highlighted mechanisms through which early marriage affects mental health due to a lack of autonomy and control over their lives. This is supported by longitudinal evidence from Northern India, which found a reciprocal relationship between early marriage and mental health; girls who marry early reported worse mental health before marriage, which proceeded to deteriorate further after marriage [149]. However, quantitative evidence from Pakistan questions the assumed association between autonomy and mental health in South Asia, highlighting that in this context the promotion of self-denial and self-sacrifice changes people’s conceptualisation of mental health relative to other settings, calling for context-specific indicators [150]. Further research considering how context- and pregnancy-specific factors intersect to influence the mental health of young and early married women is needed.

## Conclusion

We highlight the extent to which relationships within households and communities affect the health and wellbeing of early married women in South Asia. Individual-level factors, such as education and empowerment, can improve the knowledge of women and girls. However, translating this into improved care-seeking, mental health, and nutrition requires engagement at the household and community levels to ensure women and girls are being trusted and listened to.

## Supporting information

S1 Appendix Complete search strategies for each database: Ovid (MEDLINE, EMBASE, PsychINFO, and Global Health), Scopus, Global Index Medicus (WHO), CINAHL (EBSCO), Web of Science, and PROQUEST. (DOCX)

S2 Appendix Quality appraisal for high, medium, and low relevance studies, as determined using the Critical Appraisal Skills Programme (CASP) quality appraisal tool and the relevance of the study to the review question, organised by country and year of publication. (DOCX)

S3 Appendix Data extraction tables for medium and low relevance studies, organised by country and year of publication. (DOCX)

S4 Appendix Heat map of themes and subthemes, in which a darker colour representing a higher referenced theme, with supporting evidence. (DOCX)

S5 Appendix Completed PRISMA 2020 checklist addressing the reporting of review components in the title, abstract, introduction, methods, results and discussion sections of a systematic review report. (DOCX)

## References

[1] UN General Assembly. Universal Declaration of Human Rights [Internet]. 1948 [cited 2021 Jan 24]. Available from: https://www.un.org/en/universal-declaration-human-rights/

[2] UNICEF. UNICEF DATA. 2018 [cited 2021 Mar 28]. Child Marriage: Latest trends and future prospects. Available from: https://data.unicef.org/resources/child-marriage-latest-trends-and-future-prospects/

[3] Ganchimeg T, Mori R, Ota E, Koyanagi A, Gilmour S, Shibuya K, et al. Maternal and perinatal outcomes among nulliparous adolescents in low- and middle-income countries: a multi-country study. BJOG Int J Obstet Gynaecol. 2013 Dec;120(13):1622–30; discussion 1630. doi: 10.1111/1471-0528.12391 23924217

[4] Gurung R, Målqvist M, Hong Z, Poudel PG, Sunny AK, Sharma S, et al. The burden of adolescent motherhood and health consequences in Nepal. BMC Pregnancy Childbirth. 2020 May 24;20(1):318. doi: 10.1186/s12884-020-03013-8 32448326 PMC7245914

[5] Miller, Marphatia AA, Wells JC, Cortina-Borja M, Manandhar DS, Saville NM. Associations between early marriage and preterm delivery: Evidence from lowland Nepal. Am J Hum Biol. 2021;e23709. doi: 10.1002/ajhb.23709 34862821 PMC11475576

[6] Sharma V, Katz J, Mullany LC, Khatry SK, LeClerq SC, Shrestha SR, et al. Young Maternal Age and the Risk of Neonatal Mortality in Rural Nepal. Arch Pediatr Adolesc Med. 2008 Sep;162(9):828–35. doi: 10.1001/archpedi.162.9.828 18762599 PMC2535853

[7] Efevbera Y, Bhabha J, Farmer PE, Fink G. Girl child marriage as a risk factor for early childhood development and stunting. Soc Sci Med 1982. 2017;185:91–101. doi: 10.1016/j.socscimed.2017.05.027 28570927

[8] Wells JCK, Marphatia AA, Manandhar DS, Cortina-Borja M, Reid AM, Saville NS. Associations of age at marriage and first pregnancy with maternal nutritional status in Nepal. Evol Med Public Health. 2022 Jan 1;10(1):325–38. doi: 10.1093/emph/eoac025 35935708 PMC9346504

[9] Plesons M, Travers E, Malhotra A, Finnie A, Maksud N, Chalasani S, et al. Updated research gaps on ending child marriage and supporting married girls for 2020–2030. Reprod Health. 2021 Jul 20;18(1):152. doi: 10.1186/s12978-021-01176-x 34284797 PMC8290547

[10] Bicchieri C, Jiang T, Lindemans JW. A Social Norms Perspective on Child Marriage: The General Framework [Internet]. UNICEF and University of Pennsylvania; 2014 [cited 2022 Aug 2]. Available from: https://repository.upenn.edu/cgi/viewcontent.cgi?article=1012&context=pennsong

[11] Greene ME, Stiefvater E. Social and gender norms and child marriage [Internet]. ALiGN (Advancing Learning and Innovation on Gender Norms; 2019 p. 21. Available from: https://www.alignplatform.org/sites/default/files/2019-04/align_child_marriage_thinkpiece.pdf

[12] Doss CR, Meinzen-Dick R, Pereira A, Pradhan R. Women’s empowerment, extended families and male migration in Nepal: Insights from mixed methods analysis. J Rural Stud. 2022 Feb 1;90:13–25. doi: 10.1016/j.jrurstud.2022.01.003 35300181 PMC8919429

[13] Harris-Fry H, Prost A, Beaumont E, Fivian E, Mohanty S, Parida M, et al. Intrahousehold power inequalities and cooperation: Unpacking household responses to nutrition-sensitive agriculture interventions in rural India. Matern Child Nutr. 2023;e13503. doi: 10.1111/mcn.13503 36939121 PMC10262904

[14] Marphatia AA, Ambale GS, Reid AM. Women’s Marriage Age Matters for Public Health: A Review of the Broader Health and Social Implications in South Asia. Front Public Health [Internet]. 2017 Oct 18 [cited 2021 Jan 19];5. Available from: https://www.ncbi.nlm.nih.gov/pmc/articles/PMC5651255/ doi: 10.3389/fpubh.2017.00269 29094035 PMC5651255

[15] Sekine K, Hodgkin ME. Effect of child marriage on girls’ school dropout in Nepal: Analysis of data from the Multiple Indicator Cluster Survey 2014. PLOS ONE. 2017 Jul 20;12(7):e0180176. doi: 10.1371/journal.pone.0180176 28727793 PMC5519022

[16] MOHP. Nepal Demographic and Health Survey 2016. Kathmandu, Nepal; 2017.

[17] Sekine K, Carter DJ. The effect of child marriage on the utilization of maternal health care in Nepal: A cross-sectional analysis of Demographic and Health Survey 2016. PLOS ONE. 2019 Sep 19;14(9):e0222643. doi: 10.1371/journal.pone.0222643 31536591 PMC6752778

[18] Dadras O, Hazratzai M, Dadras F. The association of child marriage with morbidities and mortality among children under 5 years in Afghanistan: findings from a national survey. BMC Public Health. 2023 Jan 5;23(1):32. 36604673 10.1186/s12889-023-14977-5 PMC9817260

[19] Kidman R. Child marriage and intimate partner violence: a comparative study of 34 countries. Int J Epidemiol. 2017 Apr 1;46(2):662–75. doi: 10.1093/ije/dyw225 27733435

[20] Godha D, Hotchkiss DR, Gage AJ. Association Between Child Marriage and Reproductive Health Outcomes and Service Utilization: A Multi-Country Study From South Asia. J Adolesc Health. 2013 May 1;52(5):552–8. doi: 10.1016/j.jadohealth.2013.01.021 23608719

[21] Raj A, Boehmere U. Girl Child Marriage and Its Association With National Rates of HIV, Maternal Health, and Infant Mortality Across 97 Countries. Violence Women. 2013;19(4):536–51. doi: 10.1177/1077801213487747 23698937

[22] Subramanee SD, Agho K, Lakshmi J, Huda MN, Joshi R, Akombi-Inyang B. Child Marriage in South Asia: A Systematic Review. Int J Environ Res Public Health. 2022 Jan;19(22):15138. doi: 10.3390/ijerph192215138 36429857 PMC9691026

[23] Burgess RA, Jeffery M, Odero SA, Rose-Clarke K, Devakumar D. Overlooked and unaddressed: A narrative review of mental health consequences of child marriages. PLOS Glob Public Health. 2022 Jan 12;2(1):e0000131. doi: 10.1371/journal.pgph.0000131 36962120 PMC10021205

[24] Irani M, Roudsari RL. Reproductive and Sexual Health Consequences of Child Marriage: A Review of literature. J Midwifery Reprod Health [Internet]. 2018 Oct [cited 2022 Jan 12];(Online First). Available from: 10.22038/jmrh.2018.31627.1342

[25] Santhya KG. Early marriage and sexual and reproductive health vulnerabilities of young women: a synthesis of recent evidence from developing countries. Curr Opin Obstet Gynecol. 2011 Oct;23(5):334–9. doi: 10.1097/GCO.0b013e32834a93d2 21836504

[26] Fan S, Koski A. PROSPERO. 2020 [cited 2022 Jan 12]. The effects of child marriage on health across the globe: A systematic review of available evidence. CRD42020182652. Available from: https://www.crd.york.ac.uk/prospero/display_record.php?RecordID=182652

[27] Campbell M, Egan M, Lorenc T, Bond L, Popham F, Fenton C, et al. Considering methodological options for reviews of theory: illustrated by a review of theories linking income and health. Syst Rev. 2014 Oct 13;3(1):114. doi: 10.1186/2046-4053-3-114 25312937 PMC4208031

[28] Agnihotri RK. Linguistic diversity and marginality in South Asia. In: Handbook of education systems in South Asia. Springer; 2021. p. 1401–36.

[29] Yeung WJJ, Desai S, Jones GW. Families in Southeast and South Asia. Annu Rev Sociol. 2018;44(1):469–95.

[30] Navaneetham K, Dharmalingam A. A Review of Age Structural Transition and Demographic Dividend in South Asia: Opportunities and Challenges. J Popul Ageing. 2012 Dec 1;5(4):281–98.

[31] Parsons J, Edmeades J, Kes A, Petroni S, Sexton M, Wodon Q. Economic Impacts of Child Marriage: A Review of the Literature. Rev Faith Int Aff. 2015 Jul 3;13(3):12–22.

[32] Miller F, Saville N, Harris-Fry H, Gram L. PROSPERO. 2022 [cited 2022 Feb 10]. Influence of early marriage and early pregnancy on experiences of pregnancy in South Asia: A thematic synthesis of qualitative studies. Available from: https://www.crd.york.ac.uk/prospero/display_record.php?RecordID=304336 10.1371/journal.pgph.0002279 PMC10593245 37871001

[33] United Nations. The United Nations Statistics Division. 2008 [cited 2023 Feb 21]. Strengthening statistical capacity in support of progress towards the Internationally Agreed Development Goals in countries of South Asia. Available from: https://unstats.un.org/unsd/DA/SouthAsia/SouthAsia.htm

[34] Briscoe S, Bethel A, Rogers M. Conduct and reporting of citation searching in Cochrane systematic reviews: A cross-sectional study. Res Synth Methods. 2020;11(2):169–80. doi: 10.1002/jrsm.1355 31127978 PMC7079050

[35] CASP. Critical Appraisal Skills Programme tool for qQualitative research [Internet]. Critical Appraisal Skills Programme.; 2018. Available from: https://casp-uk.net/images/checklist/documents/CASP-Qualitative-Studies-Checklist/CASP-Qualitative-Checklist-2018_fillable_form.pdf

[36] Hannes K, Lockwood C, Pearson A. A Comparative Analysis of Three Online Appraisal Instruments’ Ability to Assess Validity in Qualitative Research. Qual Health Res. 2010 Dec 1;20(12):1736–43. doi: 10.1177/1049732310378656 20671302

[37] Long HA, French DP, Brooks JM. Optimising the value of the critical appraisal skills programme (CASP) tool for quality appraisal in qualitative evidence synthesis. Res Methods Med Health Sci. 2020 Sep 1;1(1):31–42.

[38] Barnett-Page E, Thomas J. Methods for the synthesis of qualitative research: a critical review. BMC Med Res Methodol. 2009 Aug 11;9(1):59. doi: 10.1186/1471-2288-9-59 19671152 PMC3224695

[39] Drisko JW. Qualitative research synthesis: An appreciative and critical introduction. Qual Soc Work. 2020 Jul 1;19(4):736–53.

[40] Thomas J, Harden A. Methods for the thematic synthesis of qualitative research in systematic reviews. BMC Med Res Methodol. 2008 Jul 10;8:45. doi: 10.1186/1471-2288-8-45 18616818 PMC2478656

[41] Noblit GW, Hare RD, Hare RD. Meta-Ethnography: Synthesizing Qualitative Studies. SAGE; 1988. 112 p.

[42] Lucas PJ, Baird J, Arai L, Law C, Roberts HM. Worked examples of alternative methods for the synthesis of qualitative and quantitative research in systematic reviews. BMC Med Res Methodol. 2007 Jan 15;7(1):4. doi: 10.1186/1471-2288-7-4 17224044 PMC1783856

[43] Booth A, Noyes J, Flemming K, Gerhardus A, Wahlster P, van der Wilt GJ, et al. Structured methodology review identified seven (RETREAT) criteria for selecting qualitative evidence synthesis approaches. J Clin Epidemiol. 2018 Jul;99:41–52. doi: 10.1016/j.jclinepi.2018.03.003 29548841

[44] Cameron M. On the Edge of the Auspicious: Gender and Caste in Nepal [Internet]. Champaign, Illinois: University of Illinois Press; 1998 [cited 2023 Feb 8]. Available from: https://books.google.com.np/books?hl=en&lr=&id=tpyj1w9HBrEC&oi=fnd&pg=PP11&dq=mary+cameron+1993+nepal&ots=7MGAho75c4&sig=XKVECq-n4_kiwPI6B5zK1iPQhYU&redir_esc=y#v=onepage&q=mary%20cameron%201993%20nepal&f=false

[45] Hamid S, Johansson E, Rubenson B. “Who am I? Where am I?” Experiences of married young women in a slum in Islamabad, Pakistan. BMC Public Health. 2009 Jul 28;9:265. doi: 10.1186/1471-2458-9-265 19638190 PMC2724518

[46] Maharjan B, Rishal P, Svanemyr J. Factors influencing the use of reproductive health care services among married adolescent girls in Dang District, Nepal: a qualitative study. BMC Pregnancy Childbirth. 2019 May 3;19(1):152. doi: 10.1186/s12884-019-2298-3 31053108 PMC6500073

[47] Paudel M, Javanparast S, Dasvarma G, Newman L. A qualitative study about the gendered experiences of motherhood and perinatal mortality in mountain villages of Nepal: implications for improving perinatal survival. BMC Pregnancy Childbirth. 2018 May 15;18(1):163. doi: 10.1186/s12884-018-1776-3 29764385 PMC5952814

[48] Perera D, Lund R, Swahnberg K, Schei B, Infanti JJ, Darj E, et al. ‘When helpers hurt’: women’s and midwives’ stories of obstetric violence in state health institutions, Colombo district, Sri Lanka. BMC Pregnancy Childbirth. 2018 Jun 7;18(1):211. doi: 10.1186/s12884-018-1869-z 29879946 PMC5991468

[49] Pike V, Ramage A, Bhardwaj A, Busch-Hallen J, Roche M. Family influences on health and nutrition practices of pregnant adolescents in Bangladesh. 2021 [cited 2023 Feb 8];17(Suppl 1). Available from: https://onlinelibrary.wiley.com/doi/full/10.1111/mcn.13159 10.1111/mcn.13159 PMC8269142 34241958

[50] Kamran I, Niazi R, Khan K, Abbas F. Situation analysis of reproductive health of adolescents and youth in Pakistan. Reprod Health [Internet]. 2019 Jan 1; Available from: https://knowledgecommons.popcouncil.org/departments_sbsr-rh/1019

[51] Rajbanshi S, Norhayati MN, Nik Hazlina NH. Risk perceptions among high-risk pregnant women in Nepal: a qualitative study. BMC Pregnancy Childbirth. 2021 Aug 4;21(1):539. doi: 10.1186/s12884-021-04018-7 34348703 PMC8340362

[52] Rashid SF. Emerging changes in reproductive behaviour among married adolescent girls in an urban slum in Dhaka, Bangladesh. Reprod Health Matters. 2006 May;14(27):151–9. doi: 10.1016/S0968-8080(06)27221-5 16713890

[53] Rashid SF. Human rights and reproductive health: political realities and pragmatic choices for married adolescent women living in urban slums, Bangladesh. BMC Int Health Hum Rights. 2011 Dec 16;11(3):S3. doi: 10.1186/1472-698X-11-S3-S3 22376023 PMC3287459

[54] Samandari G, Sarker BK, Grant C, Huq NL, Talukder A, Mahfuz SN, et al. Understanding individual, family and community perspectives on delaying early birth among adolescent girls: findings from a formative evaluation in rural Bangladesh. BMC Womens Health. 2020 Aug 10;20(1):169. doi: 10.1186/s12905-020-01044-z 32778096 PMC7419185

[55] Schuler SR, Bates LM, Islam F, Islam MK. The timing of marriage and childbearing among rural families in Bangladesh: choosing between competing risks. Soc Sci Med 1982. 2006 Jun;62(11):2826–37. doi: 10.1016/j.socscimed.2005.11.004 16352384

[56] Shahabuddin A, Nöstlinger C, Delvaux T, Sarker M, Delamou A, Bardají A, et al. Exploring Maternal Health Care-Seeking Behavior of Married Adolescent Girls in Bangladesh: A Social-Ecological Approach. PLOS ONE. 2017 Jan 17;12(1):e0169109. doi: 10.1371/journal.pone.0169109 28095432 PMC5240914

[57] Shahabuddin A, Delvaux T, Nöstlinger C, Sarker M, Bardají A, Sharkey A, et al. Maternal health care-seeking behaviour of married adolescent girls: A prospective qualitative study in Banke District, Nepal. PloS One. 2019;14(6):e0217968. doi: 10.1371/journal.pone.0217968 31237907 PMC6592531

[58] Ainul S, Amin S. Early marriage as a risk factor for mistimed pregnancy among married adolescents in Bangladesh. Poverty Gend Youth [Internet]. 2015 Jan 1; Available from: https://knowledgecommons.popcouncil.org/departments_sbsr-pgy/782

[59] Amin A, Bentley M. The Influence of Gender on Rural Women’s Illness Experiences and Health-Seeking Strategies for Gynaecological Symptoms. J Health Manag. 2002 Oct 1;4:229–49.

[60] Bhandari S, Joshi S. Perception and Perceived Experiences about Prevention and Consequences of Teenage Pregnancy and Childbirth among Teenage Mothers: A Qualitative Study. J Adv Acad Res. 2017 Feb 11;3:164.

[61] Bhuiya A, Aziz A, Chowdhury M. Ordeal of women for induced abortion in a rural area of Bangladesh. J Health Popul Nutr. 2001 Dec;19(4):281–90. 11855350

[62] Brunson J. Confronting maternal mortality, controlling birth in Nepal: the gendered politics of receiving biomedical care at birth. Soc Sci Med 1982. 2010 Nov;71(10):1719–27. doi: 10.1016/j.socscimed.2010.06.013 20713304

[63] Deuba K, Mainali A, Alvesson HM, Karki DK. Experience of intimate partner violence among young pregnant women in urban slums of Kathmandu Valley, Nepal: a qualitative study. BMC Womens Health. 2016 Mar 5;16(1):11. doi: 10.1186/s12905-016-0293-7 26945755 PMC4779579

[64] Gittelsohn J, Thapa M, Landman LT. Cultural factors, caloric intake and micronutrient sufficiency in rural Nepali households. Soc Sci Med. 1997 Jun 1;44(11):1739–49. doi: 10.1016/s0277-9536(96)00375-9 9178416

[65] Barr H. Marry before your house is swept away: child marriage in Bangladesh. New York, N.Y.: Human Rights Watch; 2015. 134 p.

[66] Kashyap A. No Tally of the Anguish. Hum Rights Watch [Internet]. 2009 Oct 7 [cited 2023 Feb 8]; Available from: https://www.hrw.org/report/2009/10/07/no-tally-anguish/accountability-maternal-health-care-india

[67] Barr H, Shrestha E, Thapa T. “Our time to sing and play”—child marriage in Nepal. New York, N.Y: Human Rights Watch; 2016. 123 p.

[68] Iyengar SD, Iyengar K, Martines JC, Dashora K, Deora KK. Childbirth practices in rural Rajasthan, India: implications for neonatal health and survival. J Perinatol Off J Calif Perinat Assoc. 2008 Dec;28 Suppl 2:S23–30. doi: 10.1038/jp.2008.174 19057565

[69] Kaartinen L, Diwan V. Mother and child health care in Kabul, Afghanistan with focus on the mother: women’s own perspective. Acta Obstet Gynecol Scand. 2002 Jun;81(6):491–501. doi: 10.1034/j.1600-0412.2002.810604.x 12047301

[70] Kaphle S, Hancock H, Newman LA. Childbirth traditions and cultural perceptions of safety in Nepal: critical spaces to ensure the survival of mothers and newborns in remote mountain villages. Midwifery. 2013 Oct;29(10):1173–81. doi: 10.1016/j.midw.2013.06.002 23845450

[71] Khanna R, Sheth M, Talati P, Damor K, Chauhan B. Social and economic marginalisation and sexual and reproductive health and rights of urban poor young women: a qualitative study from Vadodara, Gujarat, India. Sex Reprod Health Matters. 2021;29(2):2059898. doi: 10.1080/26410397.2022.2059898 35486102 PMC9067967

[72] Kwesiga D, Tawiah C, Imam MA, Tesega AK, Nareeba T, Enuameh YAK, et al. Barriers and enablers to reporting pregnancy and adverse pregnancy outcomes in population-based surveys: EN-INDEPTH study. Popul Health Metr. 2021 Feb 8;19(Suppl 1):15. doi: 10.1186/s12963-020-00228-x 33557858 PMC7869448

[73] Mukherjee A. Impact of Early Marriage on Women’s Autonomy, Sexual and Reproductive Health, and Mental Health. In: Dynamics of Early Marriage of Girls in Rural Uttar Pradesh: A Study into the Economic, Social and Human Rights Aspects. International Institute for Population Sciences: Thesis submitted for the degree of Doctor of Philosophy in Population Studies; 2017.

[74] Mukhopadhyay S, Ray S, Bhatia J. Mothers’ Perceptions and Attitudes towards Maternal Morbidity in Rural West Bengal: Findings from Focus Group Discussions. Indian J Gend Stud. 2004 Oct 1;11:369–87.

[75] Mumtaz Z, Salway S. Understanding gendered influences on women’s reproductive health in Pakistan: Moving beyond the autonomy paradigm. Soc Sci Med. 2009 Apr 1;68(7):1349–56. doi: 10.1016/j.socscimed.2009.01.025 19232810

[76] Mumtaz Z, Salway SM. Gender, pregnancy and the uptake of antenatal care services in Pakistan. Sociol Health Illn. 2007 Jan;29(1):1–26. doi: 10.1111/j.1467-9566.2007.00519.x 17286703

[77] Omer S, Zakar R, Zakar M, Fischer F. Influence of Social and Cultural Practices on Maternal Mortality: A Qualitative Study from South Punjab, Pakistan. 2020. 10.1186/s12978-021-01151-6 PMC8130310 34006307

[78] Nasrullah M, Zakar R, Zakar MZ, Krämer A. Girl-Child Marriage and Its Association with Morbidity and Mortality of Children under 5 Years of Age in a Nationally-Representative Sample of Pakistan. J Pediatr. 2014 Mar 1;164(3):639–46. doi: 10.1016/j.jpeds.2013.11.017 24367981

[79] Naved RT, Persson LA. Factors associated with spousal physical violence against women in Bangladesh. Stud Fam Plann. 2005 Dec;36(4):289–300. doi: 10.1111/j.1728-4465.2005.00071.x 16395946

[80] Rajbanshi S, Norhayati MN, Nik Hazlina NH. A Qualitative Study to Explore the Barriers for Nonadherence to Referral to Hospital Births by Women with High-Risk Pregnancies in Nepal. Int J Environ Res Public Health. 2021 May 28;18(11):5801. doi: 10.3390/ijerph18115801 34071394 PMC8199227

[81] Rizvi N S Khan K, Shaikh BT. Gender: shaping personality, lives and health of women in Pakistan. BMC Womens Health. 2014 Apr 1;14(1):53. doi: 10.1186/1472-6874-14-53 24690271 PMC3978051

[82] Sahoo K, Hulland K, Caruso B, Swain R, Freeman M, Panigrahi P, et al. Sanitation-related psychosocial stress: A grounded theory study of women across the life-course in Odisha, India. Soc Sci Med 1982. 2015 Jun 27;139:80–9. 10.1016/j.socscimed.2015.06.031 26164119

[83] Sengupta S, Kumar S, Choudhary U, Vaishnav Y. What About Us: A Study on the Situation of Young Married Girls. Rajasthan, India: Vikalp Sansthan; 2019.

[84] Sikder SS, Labrique AB, Ullah B, Ali H, Rashid M, Mehra S, et al. Accounts of severe acute obstetric complications in Rural Bangladesh. BMC Pregnancy Childbirth. 2011 Oct 21;11(1):76. doi: 10.1186/1471-2393-11-76 22018330 PMC3250923

[85] Sultana A, Zeeshan M, Anzak S. A Phenomenological Analysis of Rural Women’s Childbirth Preferences. SAGE Open. 2022 Mar 1;12:215824402210798.

[86] Wegelin M, Abdella D, Rehan N, Ather F, Kakal T, Kok M. Gaining insight into the magnitude of and factors influencing child marriage and teenage pregnancy and their consequences in Pakistan [Internet]. Netherlands: KIT Royal Tropical Institute; 2018 [cited 2023 Feb 14]. Available from: https://www.kit.nl/publication/gaining-insight-into-the-magnitude-of-and-factors-influencing-child-marriage-and-teenage-pregnancy-and-their-consequences-in-pakistan/

[87] Winkvist A, Akhtar HZ. God should give daughters to rich families only: attitudes towards childbearing among low-income women in Punjab, Pakistan. Soc Sci Med. 2000 Jul 1;51(1):73–81. doi: 10.1016/s0277-9536(99)00440-2 10817470

[88] Mitra N, Parasuraman S. Child Marriage & Early Motherhood Understandings from Lived Experiences of Young People [Internet]. Mumbai: Tata Institute of Social Sciences; 2015. Available from: https://feministlawarchives.pldindia.org/wp-content/uploads/CMEM-REPORT.pdf

[89] Hussain R, Khan A. Women’s perceptions and experiences of sexual violence in marital relationships and its effect on reproductive health. Health Care Women Int. 2008 May;29(5):468–83. doi: 10.1080/07399330801949541 18437595

[90] Matsuyama A. -seeking behavior of women and their families during pregnancy, delivery and postpartum period in Nepal [Internet] [Ph.D.]. [United States—Maryland]: The Johns Hopkins University; 2002 [cited 2022 May 16]. Available from: https://www.proquest.com/docview/305556565/abstract/E1EC3605A04C4026PQ/1

[91] Morrison J, Giri R, Arjyal A, Kharel C, Harris-Fry H, James P, et al. Addressing anaemia in pregnancy in rural plains Nepal: A qualitative, formative study. Matern Child Nutr. 2021 Jul;17 Suppl 1(Suppl 1):e13170. 34241951 10.1111/mcn.13170 PMC8269150

[92] Raj Baral Y, Skinner J, van Teijlingen E, Lyons K. The uptake of skilled birth attendants’ services in rural Nepal: A qualitative study. J Asian Midwives JAM. 2016;3.

[93] Roberts LR, Anderson BA, Lee JW, Montgomery SB. Grief and Women: Stillbirth in the Social Context of India. Int J Childbirth. 2012 Sep;2(3):187–98. doi: 10.1891/2156-5287.2.3.187 26594592 PMC4652937

[94] Sarojini N, Deepa V, Bhargava R, Gupta A, Badola K, Banerjee S. Interrogating Interruptions: Exploring Young Women’s Mental Health Issues [Internet]. New Delhi: Sama–Resource Group for Women and Health; 2019 [cited 2023 Feb 8]. Available from: https://samawomenshealth.in/interrogating-interruptions-exploring-young-womens-mental-health-report/

[95] Sekine K, Khadka N, Carandang RR, Ong KIC, Tamang A, Jimba M. Multilevel factors influencing contraceptive use and childbearing among adolescent girls in Bara district of Nepal: a qualitative study using the socioecological model. BMJ Open. 2021 Oct 19;11(10):e046156. doi: 10.1136/bmjopen-2020-046156 34666997 PMC8527108

[96] Simkhada B. Antenatal care uptake in nepal: barriers and opportunities [Internet] [Ph.D.]. [Scotland]: University of Aberdeen (United Kingdom); 2011 [cited 2022 May 20]. Available from: https://www.proquest.com/docview/1779949629/4950C15D94B4980PQ/1

[97] Alam A, Rasheed S, Khan NUZ, Sharmin T, Huda TM, Arifeen SE, et al. How can formative research inform the design of an iron-folic acid supplementation intervention starting in first trimester of pregnancy in Bangladesh? BMC Public Health. 2015 Apr 12;15:374. doi: 10.1186/s12889-015-1697-2 25887449 PMC4425912

[98] Bhattacharjya H, Paul DP, Rakshit AK. Teenage pregnancies, practices, and utilization of RCH services by the tribal and nontribal population of West and South Tripura districts: A mixed method study. J Fam Med Prim Care. 2021 Aug;10(8):3034–9. doi: 10.4103/jfmpc.jfmpc_399_21 34660443 PMC8483111

[99] Christou A, Alam A, Sadat Hofiani SM, Rasooly MH, Mubasher A, Rashidi MK, et al. Understanding pathways leading to stillbirth: The role of care-seeking and care received during pregnancy and childbirth in Kabul province, Afghanistan. Women Birth J Aust Coll Midwives. 2020 Nov;33(6):544–55. doi: 10.1016/j.wombi.2020.02.012 32094034

[100] Ali SA, Feroz A, Abbasi Z, Ali SA, Allana A, Hambidge KM, et al. Perceptions of women, their husbands and healthcare providers about anemia in rural Pakistan: Findings from a qualitative exploratory study. PLOS ONE. 2021 Apr 27;16(4):e0249360. doi: 10.1371/journal.pone.0249360 33905421 PMC8078764

[101] Arnold R, van Teijlingen E, Ryan K, Holloway I. Villains or victims? An ethnography of Afghan maternity staff and the challenge of high quality respectful care | BMC Pregnancy and Childbirth | Full Text. BMC Pregnancy Childbirth [Internet]. 2019 [cited 2023 Feb 14];19(307). Available from: https://bmcpregnancychildbirth.biomedcentral.com/articles/10.1186/s12884-019-2420-6 10.1186/s12884-019-2420-6 PMC6708168 31443691

[102] Awasthi S, Verma T, Agarwal M. Danger signs of neonatal illnesses: perceptions of caregivers and health workers in northern India. Bull World Health Organ [Internet]. 2006 [cited 2023 Feb 14];84(10). Available from: https://apps.who.int/iris/handle/10665/269756 doi: 10.2471/blt.05.029207 17128362 PMC2627499

[103] Barua A. Role of Mothers-in-Law in Young Women’s Reproductive Health: Evidence from Intervention Research in Rural Maharashtra, India. India: Foundation for Research in Health Systems (FRHS);

[104] Barua M, Van Driel F, Jansen W. Tuberculosis and the sexual and reproductive lives of women in Bangladesh. PloS One. 2018;13(7):e0201134. doi: 10.1371/journal.pone.0201134 30024977 PMC6053219

[105] Bhatia V, Nayak R, Bhattacharjee S, Mahajan P, Parida S. Perception and Health Seeking Behaviour of People Regarding Anaemia: An Experience from Odisha in Eastern India. 2021.

[106] Biswas A, Doraiswamy S, Abdullah ASM, Purno NH, Rahman F, Halim MA. Exploring the perceptions, practices and challenges to maternal and newborn health care among the underprivileged teagarden community in Bangladesh: a qualitative study. Sex Reprod Health Matters. 2020 Dec;28(1):1758443. doi: 10.1080/26410397.2020.1758443 32524899 PMC7887958

[107] CARE. The cultural context of child marriage in Nepal and Bangladesh: findings from CARE’s Tipping Point project [Internet]. 2016 [cited 2022 Jul 20]. Available from: https://www.girlsnotbrides.org/learning-resources/resource-centre/care-tipping-point-project-nepal-bangladesh/

[108] Chorghade G, Barker M, Kanade S, Fall C. Why are rural Indian women so thin? Findings from a village in Maharashtra. Public Health Nutr. 2006 Feb;9(1):9–18. doi: 10.1079/phn2005762 16480528 PMC2655053

[109] Doke PP, Gothankar JS, Pore PD, Palkar SH, Chutke AP, Patil AV, et al. Meager Perception of Preconception Care Among Women Desiring Pregnancy in Rural Areas: A Qualitative Study Using Focus Group Discussions. Front Public Health. 2021;9:689820. doi: 10.3389/fpubh.2021.689820 34722433 PMC8553982

[110] Eshan S, Sattar T, Nisar R. Domestic violence against pregnant women and its effects on their reproductive health -. Rawal Med J. 2021;46(1):180–4.

[111] Gopichandran V, Subramaniam S, Kalsingh MJ. Psycho-social impact of stillbirths on women and their families in Tamil Nadu, India–a qualitative study. BMC Pregnancy Childbirth. 2018 Apr 20;18(1):109. doi: 10.1186/s12884-018-1742-0 29678157 PMC5910626

[112] Mahato S. Causes and Consequences of Child Marriage: A Perspective. Int J Sci Eng Res. 2016 Jul 25;7:698–702.

[113] Mayra K, Matthews Z, Padmadas SS. Why do some health care providers disrespect and abuse women during childbirth in India? Women Birth J Aust Coll Midwives. 2022 Feb;35(1):e49–59. doi: 10.1016/j.wombi.2021.02.003 33678563

[114] Raj A, Gomez CS, Silverman JG. Multisectorial Afghan perspectives on girl child marriage: foundations for change do exist in Afghanistan. Violence Women. 2014 Dec;20(12):1489–505. doi: 10.1177/1077801211403288 21450680

[115] Rajbanshi S, Norhayati MN, Nik Hazlina NH. Perceptions of Good-Quality Antenatal Care and Birthing Services among Postpartum Women in Nepal. Int J Environ Res Public Health. 2021 Jun 26;18(13):6876. doi: 10.3390/ijerph18136876 34206868 PMC8297004

[116] Ramakrishnan U, Lowe A, Vir S, Kumar S, Mohanraj R, Chaturvedi A, et al. Public health interventions, barriers, and opportunities for improving maternal nutrition in India. Food Nutr Bull. 2012 Jun;33(2 Suppl):S71–92. doi: 10.1177/15648265120332S105 22913108

[117] Rasweth S, Nisha B. The unspoken plight of married adolescent girls in rural Tamil Nadu: Narrative summary on unmet sexual and reproductive health needs and barriers. Indian J Community Health. 2022;34(3):439–43.

[118] Sarkar A, Kharmujai OM, Lynrah W, Suokhrie NU. Factors influencing the place of delivery in rural Meghalaya, India: A qualitative study. J Fam Med Prim Care. 2018;7(1):98–103. doi: 10.4103/jfmpc.jfmpc_45_17 29915741 PMC5958601

[119] Seth R, Bose V, Qaiyum Y, Chandrashekhar R, Kansal S, Taneja I, et al. Social Determinants of Child Marriage in Rural India. Ochsner J. 2018;18(4):390–4. doi: 10.31486/toj.18.0104 30559625 PMC6292470

[120] Shah R, Rehfuess EA, Paudel D, Maskey MK, Delius M. Barriers and facilitators to institutional delivery in rural areas of Chitwan district, Nepal: a qualitative study. Reprod Health. 2018 Jun 20;15(1):110. doi: 10.1186/s12978-018-0553-0 29925398 PMC6011343

[121] Vlassoff C, Rao S, Vishnu Lale S. Can conditional cash transfers promote delayed childbearing? Evidence from the ‘Second Honeymoon Package’ in rural Maharashtra, India. Asian Popul Stud. 2017 Jan 2;13(1):86–100.

[122] Wyatt S, Ostbye T, De Silva V, Lakmali P, Long Q. Predictors and occurrence of antenatal depressive symptoms in Galle, Sri Lanka: a mixed-methods cross-sectional study. BMC Pregnancy Childbirth. 2021 Nov 10;21(1):758. doi: 10.1186/s12884-021-04239-w 34758774 PMC8578523

[123] Page MJ, McKenzie JE, Bossuyt PM, Boutron I, Hoffmann TC, Mulrow CD, et al. The PRISMA 2020 statement: an updated guideline for reporting systematic reviews. BMJ. 2021 Mar 29;372:n71. doi: 10.1136/bmj.n71 33782057 PMC8005924

[124] Nguyen PH, Scott S, Neupane S, Tran LM, Menon P. Social, biological, and programmatic factors linking adolescent pregnancy and early childhood undernutrition: a path analysis of India’s 2016 National Family and Health Survey. Lancet Child Adolesc Health. 2019 Jul;3(7):463–73. doi: 10.1016/S2352-4642(19)30110-5 31105055 PMC6558962

[125] Habib SS, Jamal WZ, Zaidi SMA, Siddiqui JUR, Khan HM, Creswell J, et al. Barriers to Access of Healthcare Services for Rural Women-Applying Gender Lens on TB in a Rural District of Sindh, Pakistan. Int J Environ Res Public Health. 2021 Sep 26;18(19):10102. doi: 10.3390/ijerph181910102 34639403 PMC8508279

[126] Adhikari R. Effect of Women’s autonomy on maternal health service utilization in Nepal: a cross sectional study. BMC Womens Health. 2016 May 13;16:26. doi: 10.1186/s12905-016-0305-7 27177683 PMC4867085

[127] Batura N, Poupakis S, Das S, Bapat U, Alcock G, Skordis J, et al. Factors associated with women’s healthcare decision-making during and after pregnancy in urban slums in Mumbai, India: a cross-sectional analysis. BMC Public Health. 2022 Dec;22(1):1–7. 35418068 10.1186/s12889-022-13216-7 PMC9009007

[128] Agarwal B.”Bargaining” and Gender Relations: Within and Beyond the Household. Fem Econ. 1997 Jan 1;3(1):1–51.

[129] Lavender T, Bedwell C, Blaikie K, Danna VA, Sutton C, Kasengele CT, et al. Journey of vulnerability: a mixed-methods study to understand intrapartum transfers in Tanzania and Zambia. BMC Pregnancy Childbirth. 2020 May 14;20(1):292. doi: 10.1186/s12884-020-02996-8 32408871 PMC7222428

[130] Actis Danna V, Bedwell C, Wakasiaka S, Lavender T. Utility of the three-delays model and its potential for supporting a solution-based approach to accessing intrapartum care in low- and middle-income countries. A qualitative evidence synthesis. Glob Health Action. 2020 Dec 31;13(1):1819052. doi: 10.1080/16549716.2020.1819052 33040697 PMC7580724

[131] Pokharel A, Pokharel SD. Women’s involvement in decision-making and receiving husbands’ support for their reproductive healthcare: a cross-sectional study in Lalitpur, Nepal. Int Health. 2023 Jan 3;15(1):67–76. doi: 10.1093/inthealth/ihac034 35639520 PMC9808508

[132] Diamond-Smith N, Puri M, Neuhaus J, Weiser S, Kadiyala S. Do changes in women’s household status in Nepal improve access to food and nutrition? Matern Child Nutr. 2022;18(3):e13374. doi: 10.1111/mcn.13374 35615780 PMC9218303

[133] Aryal KK, Sharma SK, Khanal MN, Bista B, Sharma SL, Kafle S, et al. Maternal Health Care in Nepal: Trends and Determinants. 2019.

[134] Ghose B, Feng D, Tang S, Yaya S, He Z, Udenigwe O, et al. Women’s decision-making autonomy and utilisation of maternal healthcare services: results from the Bangladesh Demographic and Health Survey. BMJ Open. 2017 Sep 1;7(9):e017142. doi: 10.1136/bmjopen-2017-017142 28882921 PMC5595179

[135] Jejeebhoy SJ, Sathar ZA. Women’s Autonomy in India and Pakistan: The Influence of Religion and Region. Popul Dev Rev. 2001;27(4):687–712.

[136] Delprato M, Akyeampong K, Sabates R, Hernandez-Fernandez J. On the impact of early marriage on schooling outcomes in Sub-Saharan Africa and South West Asia. Int J Educ Dev. 2015 Sep 1;44:42–55.

[137] Del Franco N. Aspirations and self‐hood: exploring the meaning of higher secondary education for girl college students in rural Bangladesh. Comp J Comp Int Educ. 2010 Mar 1;40(2):147–65.

[138] Marphatia AA, Saville NM, Amable GS, Manandhar DS, Cortina-Borja M, Wells JC, et al. How Much Education Is Needed to Delay Women’s Age at Marriage and First Pregnancy? Front Public Health. 2020;7:396. doi: 10.3389/fpubh.2019.00396 31993411 PMC6964653

[139] Marphatia AA, Saville NM, Manandhar DS, Cortina-Borja M, Wells JCK, Reid AM. The role of education in child and adolescent marriage in rural lowland Nepal. J Biosoc Sci. 2023 Mar;55(2):275–91. doi: 10.1017/S0021932022000074 35361308

[140] Scott S, Nguyen PH, Neupane S, Pramanik P, Nanda P, Bhutta ZA, et al. Early marriage and early childbearing in South Asia: trends, inequalities, and drivers from 2005 to 2018. Ann N Y Acad Sci [Internet]. 2020 [cited 2021 Mar 8];n/a(n/a). Available from: http://nyaspubs.onlinelibrary.wiley.com/doi/abs/10.1111/nyas.14531 33258141 10.1111/nyas.14531 PMC8247060

[141] Rahman M, Saha P, Uddin J. Associations of antenatal care visit with utilization of institutional delivery care services in Afghanistan: intersections of education, wealth, and household decision-making autonomy. BMC Pregnancy Childbirth. 2022 Mar 26;22(1):255. doi: 10.1186/s12884-022-04588-0 35346100 PMC8961925

[142] Rohner RP, Chaki-Sircar M. Women and children in a Bengali village [Internet]. Published for University of Connecticut, University Press of New England; 1988 [cited 2023 Mar 21]. Available from: https://ehrafworldcultures.yale.edu/cultures/aw69/documents/019

[143] Harris-Fry H, Shrestha N, Costello A, Saville NM. Determinants of intra-household food allocation between adults in South Asia–a systematic review. Int J Equity Health. 2017 Jun 21;16(1):107. doi: 10.1186/s12939-017-0603-1 28637477 PMC5480108

[144] Acharya DR, Bell JS, Simkhada P, van Teijlingen ER, Regmi PR. Women’s autonomy in household decision-making: a demographic study in Nepal. Reprod Health. 2010 Jul 15;7:15. doi: 10.1186/1742-4755-7-15 20630107 PMC2914657

[145] Bhatta DN. Involvement of males in antenatal care, birth preparedness, exclusive breast feeding and immunizations for children in Kathmandu, Nepal. BMC Pregnancy Childbirth. 2013 Jan 16;13:14. doi: 10.1186/1471-2393-13-14 23324410 PMC3558464

[146] Bloom SS, Wypij D, Das Gupta M. Dimensions of women’s autonomy and the influence on maternal health care utilization in a North Indian city. Demography. 2001 Feb 1;38(1):67–78. doi: 10.1353/dem.2001.0001 11227846

[147] Uddin J, Pulok MH, Johnson RB, Rana J, Baker E. Association between child marriage and institutional delivery care services use in Bangladesh: intersections between education and place of residence. Public Health. 2019 Jun;171:6–14. doi: 10.1016/j.puhe.2019.03.014 31071578

[148] Raj A, Johns NE, Bhan N, Silverman JG, Lundgren R. Effects of Gender Role Beliefs on Social Connectivity and Marital Safety: Findings From a Cross-Sectional Study Among Married Adolescent Girls in India. J Adolesc Health Off Publ Soc Adolesc Med. 2021 Dec;69(6S):S65–73. doi: 10.1016/j.jadohealth.2021.07.030 34809903

[149] Aggarwal S, Francis KL, Dashti SG, Patton G. Child marriage and the mental health of adolescent girls: a longitudinal cohort study from Uttar Pradesh and Bihar, India. Lancet Reg Health—Southeast Asia. 2023;8:100102. doi: 10.1016/j.lansea.2022.100102 37384140 PMC10306007

[150] Ali SM, Haq R ul. Women’s Autonomy and Happiness: The Case of Pakistan. Pak Dev Rev. 2006;45(1):121–36.

