# Supplementary material for: “Can’t live willingly”: A thematic synthesis of qualitative evidence exploring how early marriage and early pregnancy affect experiences of pregnancy in South Asia
Source: PLOS Glob Public Health. 2023 Oct 23;3(10):e0002279. doi: 10.1371/journal.pgph.0002279 (PMC10593245; doi:10.1371/journal.pgph.0002279)
Supplement: S1 Appendix — (DOCX) [file pgph.0002279.s001.docx]

## Ovid - MEDLINE, EMBASE, PsychINFO AND Global Health

1

(early OR young OR younger OR youth OR adolescen* OR juvenile OR matur* OR teen* OR puberty* OR menarche OR old OR older OR adult* OR bride OR bridal OR cohabit* OR "child adj3 marriage" OR marri* OR empower*).mp.

2

(pregnan* OR "child birth*" OR childbirth* OR obstetric* OR prenatal* OR "pre natal" OR antenatal OR "ante natal" OR ANC OR gestation* OR miscarriage* OR stillbirth* OR "still birth*" OR "family planning" OR contracept* OR preconception OR "pre conception" OR reproductive OR fertility OR infertility).mp.

3

((Nepal/ OR India/ OR Afghanistan/ OR Bhutan/ OR Maldives/ OR Pakistan/ OR Sri Lanka/ OR Bangladesh/) OR ("South Asia*" OR nepal* OR afghanistan* OR bhutan* OR india* OR maldiv* OR pakistan* OR "Sri Lanka*" OR Bangladesh*).mp.)

4

( qualitativ* OR "focus group*" OR fgd OR "grounded theor*" OR "thematic analys*" OR "mixed methods" OR fieldwork OR (field adj3 (work OR study OR studies OR research OR note*)) OR "key informant*" OR anthropolog* OR "participant* observation*" OR "observation* method*" OR "constant comparative method*" OR "textual analys*" OR ethnograph* OR "case stud*" OR "participatory adj4 action" OR (("Semi structured" OR semistructured OR unstructured OR informal OR "in depth" OR indepth OR cognitive OR focus* OR "face to face" OR structured OR guide OR guides OR tape* ) adj5 (interview* OR discussion* OR questionnaire* OR account)) OR (Analysis AND (conversation* OR discourse* OR documentary OR "key informant*" OR Narrative)) ).mp.

5

1 and 2 and 3 and 4

## Scopus

TITLE-ABS-KEY (early OR young OR younger OR youth OR adolescen* OR juvenile OR matur* OR teen* OR puberty* OR menarche OR old OR older OR adult* OR bride OR bridal OR cohabit OR (child w/2 marriage) OR marri* OR empower*)

AND

TITLE-ABS-KEY (pregnan* OR "child birth*" OR childbirth* OR obstetric* OR prenatal* OR "pre natal" OR antenatal OR "ante natal" OR ANC OR gestation* OR miscarriage* OR stillbirth* OR "still birth*" OR "family planning" OR contracept* OR preconception OR "pre conception" OR reproductive OR fertility OR infertility)

AND

TITLE"South Asia*" OR nepal* OR afghanistan* OR bhutan* OR india* OR maldiv* OR pakistan* OR "Sri Lanka*" OR Bangladesh*) -ABS-KEY("South Asia*" OR nepal* OR afghanistan* OR bhutan* OR india* OR maldiv* OR pakistan* OR "Sri Lanka*" OR Bangladesh*)

AND

TITLE-ABS-KEY ( qualitativ* OR "focus group*" OR fgd OR "grounded theor*" OR "thematic analys*" OR "mixed methods" OR fieldwork OR (field W/2 (work OR study OR studies OR research OR note*)) OR "key informant*" OR anthropolog* OR "participant* observation*" OR "observation* method*" OR "constant comparative method*" OR "textual analys*" OR ethnograph* OR "case stud*" OR "participatory W/3 action" OR (("Semi structured" OR semistructured OR unstructured OR informal OR "in depth" OR indepth OR cognitive OR focus* OR "face to face" OR structured OR guide OR guides OR tape* ) w/4 (interview* OR discussion* OR questionnaire* OR account)) OR (Analysis AND (conversation* OR discourse* OR documentary OR "key informant*" OR Narrative)) )

## Global Index Medicus (WHO)

tw:(early OR young OR younger OR youth OR adolescen* OR juvenile OR matur* OR teen* OR puberty* OR menarche OR old OR older OR adult* OR bride OR bridal OR cohabit* OR ("child marriage") OR marri* OR empower*)

AND

tw:(pregnan* OR "child birth*" OR childbirth* OR obstetric* OR prenatal* OR "pre natal" OR antenatal OR "ante natal" OR ANC OR gestation* OR miscarriage* OR stillbirth* OR "still birth*" OR "family planning" OR contracept* OR preconception OR "pre conception" OR reproductive OR fertility OR infertility OR "pre eclampsia" OR preeclampsia OR anemi*)

AND

tw:("South Asia*" OR nepal* OR afghanistan* OR bhutan* OR india* OR maldiv* OR pakistan* OR "Sri Lanka*" OR Bangladesh*)

AND

tw: ( qualitativ* OR "focus group*" OR fgd OR "grounded theor*" OR "thematic analys*" OR "mixed methods" OR fieldwork OR (field W/2 (work OR study OR studies OR research OR note*)) OR "key informant*" OR anthropolog* OR "participant* observation*" OR "observation* method*" OR "constant comparative method*" OR "textual analys*" OR ethnograph* OR "case stud*" OR (participatory AND action) OR (("Semi structured" OR semistructured OR unstructured OR informal OR "in depth" OR indepth OR cognitive OR focus* OR "face to face" OR structured OR guide OR guides OR tape* ) AND (interview* OR discussion* OR questionnaire* OR account)) OR (Analysis AND (conversation* OR discourse* OR documentary OR "key informant*" OR Narrative)) )

## CINAHL (EBSCO)

UNDERGO 2 SEARCHES (as the qualitative limiter is bad)

1.

With predetermined qualitative limiters

(early OR young OR younger OR youth OR adolescen* OR juvenile OR matur* OR teen* OR puberty* OR menarche OR old OR older OR adult* OR bride OR bridal OR cohabit* OR "child adj3 marriage" OR marri* OR empower* OR (MH "Marriage") OR (MH "Adolescence"))

AND

(pregnan* OR "child birth*" OR childbirth* OR obstetric* OR prenatal* OR "pre natal" OR antenatal OR "ante natal" OR ANC OR gestation* OR miscarriage* OR stillbirth* OR "still birth*" OR "family planning" OR contracept* OR preconception OR "pre conception" OR reproductive OR fertility OR infertility OR (MH "Pregnancy in Adolescence") )

AND

( ( ( "South Asia*" OR nepal* OR afghanistan* OR bhutan* OR india* OR maldiv* OR pakistan* OR "Sri Lanka*" OR Bangladesh*) OR ((MH "Asia, Western") NOT (MH "Middle East")) OR (MH "Nepal") OR (MH "Bangladesh") OR (MH "Bhutan") OR (MH "India") OR (MH "Pakistan") OR (MH "Sri Lanka") OR (MH "Afghanistan") )

[WITH Limiters - Clinical Queries: Qualitative -Best Balance]

1. Own qualitative search terminology

(early OR young OR younger OR youth OR adolescen* OR juvenile OR matur* OR teen* OR puberty* OR menarche OR old OR older OR adult* OR bride OR bridal OR cohabit* OR "child adj3 marriage" OR marri* OR empower* OR (MH "Marriage") OR (MH "Adolescence"))

AND

(pregnan* OR "child birth*" OR childbirth* OR obstetric* OR prenatal* OR "pre natal" OR antenatal OR "ante natal" OR ANC OR gestation* OR miscarriage* OR stillbirth* OR "still birth*" OR "family planning" OR contracept* OR preconception OR "pre conception" OR reproductive OR fertility OR infertility OR (MH "Pregnancy in Adolescence") )

AND

( ( ( "South Asia*" OR nepal* OR afghanistan* OR bhutan* OR india* OR maldiv* OR pakistan* OR "Sri Lanka*" OR Bangladesh*) OR ((MH "Asia, Western") NOT (MH "Middle East")) OR (MH "Nepal") OR (MH "Bangladesh") OR (MH "Bhutan") OR (MH "India") OR (MH "Pakistan") OR (MH "Sri Lanka") OR (MH "Afghanistan") )

AND

(( qualitativ* OR "focus group*" OR fgd OR "grounded theor*" OR "thematic analys*" OR "mixed methods" OR fieldwork OR (field adj3 (work OR study OR studies OR research OR note*)) OR "key informant*" OR anthropolog* OR "participant* observation*" OR "observation* method*" OR "constant comparative method*" OR "textual analys*" OR ethnograph* OR "case stud*" OR "participatory adj4 action" OR (("Semi structured" OR semistructured OR unstructured OR informal OR "in depth" OR indepth OR cognitive OR focus* OR "face to face" OR structured OR guide OR guides OR tape* ) adj5 (interview* OR discussion* OR questionnaire* OR account)) OR (Analysis AND (conversation* OR discourse* OR documentary OR "key informant*" OR Narrative)) ) OR (MH "Qualitative Studies") )

➔ COMBINE WITH OR

## Web of Science

(

(TI=( early OR young OR younger OR youth OR adolescen* OR juvenile OR matur* OR teen* OR puberty* OR menarche OR old OR older OR adult* OR bride OR bridal OR cohabit* OR "child NEAR/3 marriage" OR marri* OR empower*)) OR

(AB=( early OR young OR younger OR youth OR adolescen* OR juvenile OR matur* OR teen* OR puberty* OR menarche OR old OR older OR adult* OR bride OR bridal OR cohabit* OR "child NEAR/3 marriage" OR marri* OR empower*)) OR

(AK=( early OR young OR younger OR youth OR adolescen* OR juvenile OR matur* OR teen* OR puberty* OR menarche OR old OR older OR adult* OR bride OR bridal OR cohabit* OR "child NEAR/3 marriage" OR marri* OR empower*))

)

AND

(

(TI=(pregnan* OR "child birth*" OR childbirth* OR obstetric* OR prenatal* OR "pre natal" OR antenatal OR "ante natal" OR ANC OR gestation* OR miscarriage* OR stillbirth* OR "still birth*" OR "family planning" OR contracept* OR preconception OR "pre conception" OR reproductive OR fertility OR infertility)) OR

(AB=(pregnan* OR "child birth*" OR childbirth* OR obstetric* OR prenatal* OR "pre natal" OR antenatal OR "ante natal" OR ANC OR gestation* OR miscarriage* OR stillbirth* OR "still birth*" OR "family planning" OR contracept* OR preconception OR "pre conception" OR reproductive OR fertility OR infertility)) OR

(AK=(pregnan* OR "child birth*" OR childbirth* OR obstetric* OR prenatal* OR "pre natal" OR antenatal OR "ante natal" OR ANC OR gestation* OR miscarriage* OR stillbirth* OR "still birth*" OR "family planning" OR contracept* OR preconception OR "pre conception" OR reproductive OR fertility OR infertility))

)

AND

(

(TI=( qualitativ* OR "focus group*" OR fgd OR "grounded theor*" OR "thematic analys*" OR "mixed methods" OR fieldwork OR (field NEAR/3 (work OR study OR studies OR research OR note*)) OR "key informant*" OR anthropolog* OR "participant* observation*" OR "observation* method*" OR "constant comparative method*" OR "textual analys*" OR ethnograph* OR "case stud*" OR "participatory adj4 action" OR (("Semi structured" OR semistructured OR unstructured OR informal OR "in depth" OR indepth OR cognitive OR focus* OR "face to face" OR structured OR guide OR guides OR tape* ) NEAR/4 (interview* OR discussion* OR questionnaire* OR account)) OR (Analysis AND (conversation* OR discourse* OR documentary OR "key informant*" OR Narrative)))) OR

(AB=(qualitativ* OR "focus group*" OR fgd OR "grounded theor*" OR "thematic analys*" OR "mixed methods" OR fieldwork OR (field NEAR/3 (work OR study OR studies OR research OR note*)) OR "key informant*" OR anthropolog* OR "participant* observation*" OR "observation* method*" OR "constant comparative method*" OR "textual analys*" OR ethnograph* OR "case stud*" OR "participatory adj4 action" OR (("Semi structured" OR semistructured OR unstructured OR informal OR "in depth" OR indepth OR cognitive OR focus* OR "face to face" OR structured OR guide OR guides OR tape* ) NEAR/4 (interview* OR discussion* OR questionnaire* OR account)) OR (Analysis AND (conversation* OR discourse* OR documentary OR "key informant*" OR Narrative))) ) OR

(AK=( qualitativ* OR "focus group*" OR fgd OR "grounded theor*" OR "thematic analys*" OR "mixed methods" OR fieldwork OR (field NEAR/3 (work OR study OR studies OR research OR note*)) OR "key informant*" OR anthropolog* OR "participant* observation*" OR "observation* method*" OR "constant comparative method*" OR "textual analys*" OR ethnograph* OR "case stud*" OR "participatory adj4 action" OR (("Semi structured" OR semistructured OR unstructured OR informal OR "in depth" OR indepth OR cognitive OR focus* OR "face to face" OR structured OR guide OR guides OR tape* ) NEAR/4 (interview* OR discussion* OR questionnaire* OR account)) OR (Analysis AND (conversation* OR discourse* OR documentary OR "key informant*" OR Narrative)))

)

AND

(ALL =( "South Asia*" OR nepal* OR afghanistan* OR bhutan* OR india* OR maldiv* OR pakistan* OR "Sri Lanka*" OR Bangladesh*) )

## PROQUEST

ab((early OR young OR younger OR youth OR adolescen* OR juvenile OR matur* OR teen* OR puberty* OR menarche OR old OR older OR adult* OR bride OR bridal OR cohabit* OR "child marriage" OR marri* OR empower*))

AND

ab((pregnan* OR “child birth*” OR childbirth* OR obstetric* OR prenatal* OR “pre natal” OR antenatal OR “ante natal” OR ANC OR gestation* OR miscarriage* OR stillbirth* OR “still birth*” OR “family planning” OR contracept* OR preconception OR “pre conception” OR reproductive OR fertility OR infertility))

AND

ab((“South Asia*” OR akis* OR akistanan* OR akist* OR india* OR maldiv* OR akistan* OR “Sri Lanka*” OR Bangladesh*))

AND ab((qualitativ* OR "focus group*" OR fgd OR "grounded theor*" OR "thematic analys*" OR "mixed methods" OR fieldwork OR (field NEAR/3 (work OR study OR studies OR research OR note*)) OR "key informant*" OR anthropolog* OR "participant* observation*" OR "observation* method*" OR "constant comparative method*" OR "textual analys*" OR ethnograph* OR "case stud*" OR "participatory adj4 action" OR (("Semi structured" OR semistructured OR unstructured OR informal OR "in depth" OR indepth OR cognitive OR focus* OR "face to face" OR structured OR guide OR guides OR tape* ) NEAR/4 (interview* OR discussion* OR questionnaire* OR account)) OR (Analysis AND (conversation* OR discourse* OR documentary OR "key informant*" OR Narrative))))

## Key organisations whose websites were hand-searched

United Nations Population Fund (UNFPA), United Nations Children's Fund (UNICEF), Plan International, The International Centre for Research on Women, World Health Organisation (WHO), Care International, Human Rights Watch, Girls Not Brides, ActionAid, Save the Children, American Jewish World Service, Population Council, UK Department for International Development, KIT Royal Tropical Institute, Action Aid, OXFAM, and the World Bank.
