# Supplementary material for: “Can’t live willingly”: A thematic synthesis of qualitative evidence exploring how early marriage and early pregnancy affect experiences of pregnancy in South Asia
Source: PLOS Glob Public Health. 2023 Oct 23;3(10):e0002279. doi: 10.1371/journal.pgph.0002279 (PMC10593245; doi:10.1371/journal.pgph.0002279)
Supplement: S2 Appendix — (DOCX) [file pgph.0002279.s002.docx]

| S2 Appendix. i) Quality appraisal for high relevance studies, as determined using the CASP quality appraisal tool and the relevance of the study to the review question, organised by country and year of publication | | | | | | | | | | | | | | | | |
| --- | --- | --- | --- | --- | --- | --- | --- | --- | --- | --- | --- | --- | --- | --- | --- | --- |
| Author (Year) - Country | Title | Was there a clear statement of the aims of the research? | | Is a qualitative methodology appropriate? | Was the research design appropriate to address the aims of the research? | | Was the recruitment strategy appropriate to the aims of the research? | Was the data collected in a way that addressed the research issue? | Has the relationship between researcher and participants been adequately considered? | Have ethical issues been taken into consideration? | Are the study’s theoretical underpinnings clear, consistent and conceptually coherent? | Was the data analysis sufficiently rigorous? | Is there a clear statement of findings? | How valuable is the research? | How valuable is this research to this review? |  |
| Rajbanshi (2021) - Nepal | Risk perceptions among high-risk pregnant women in Nepal: a qualitative study | Yes | Yes | | | Somewhat | Somewhat | Yes | No | Yes | Somewhat | Yes | Yes | Somewhat valuable | High |  |
| Maharjan (2019) - Nepal | Factors influencing the use of reproductive health care services among married adolescent girls in Dang District, Nepal: A qualitative study | Yes | Yes | | | Yes | Yes | Somewhat | Somewhat | Yes | No | Somewhat | Yes | Valuable | High |  |
| Paudel (2018) - Nepal | A qualitative study about the gendered experiences of motherhood and perinatal mortality in mountain villages of Nepal: Implications for improving perinatal survival | Yes | Yes | | | Yes | Somewhat | Somewhat | Somewhat | Yes | Yes | Yes | Yes | Valuable | High |  |
| Shahabuddin (2018) - Nepal | Maternal health care-seeking behaviour of married adolescent girls: A prospective qualitative study in Banke District, Nepal | Yes | Yes | | | Yes | Yes | Yes | Somewhat | Yes | Yes | Yes | Yes | Valuable | High |  |
| Cameron (1993) - Nepal | On the edge of the auspicious: The practice and meaning of gender and caste in rural Nepal's low-caste households and society. | Yes | Yes | | | Yes | Can't tell | Yes | No | No | Yes | Somewhat | Yes | Valuable | High |  |

|  |  |  |  |  |  |  |  |  |  |  |  |  |  |
| --- | --- | --- | --- | --- | --- | --- | --- | --- | --- | --- | --- | --- | --- |
| Pike (2021) - Bangladesh | Family influences on health and nutrition practices of pregnant adolescents in Bangladesh | Yes | Yes | Yes | Somewhat | Yes | Somewhat | Somewhat | Yes | Yes | Yes | Valuable | High |
| Samandari (2020) - Bangladesh | Understanding individual, family and community perspectives on delaying early birth among adolescent girls: Findings from a formative evaluation in rural Bangladesh | Yes | Somewhat | Yes | Yes | Yes | Somewhat | Yes | No | Yes | Yes | Valuable | High |
| Shahabuddin (2017) - Bangladesh | Exploring maternal health care-seeking behavior of married adolescent girls in Bangladesh: A social-ecological approach | Yes | Yes | Yes | Yes | Somewhat | No | Yes | Yes | Yes | Yes | Valuable | High |
| Rashid (2011) - Bangladesh | Human rights and reproductive health: Political realities and pragmatic choices for married adolescent women living in urban slums, Bangladesh | Somewhat | Somewhat | Yes | Yes | Yes | Somewhat | Yes | Somewhat | Can’t tell | Somewhat | Somewhat valuable | High |
| Rashid (2006) - Bangladesh | Emerging Changes in Reproductive Behaviour among Married Adolescent Girls in an Urban Slum in Dhaka, Bangladesh | Yes | Yes | Yes | Yes | Somewhat | No | No | No | Can’t tell | Yes | Somewhat valuable | High |
| Schuler (2006) - Bangladesh | The timing of marriage and childbearing among rural families in Bangladesh: Choosing between competing risks | Yes | Yes | Yes | Somewhat | Yes | Somewhat | No | No | Somewhat | Somewhat | Valuable | High |

|  |  |  |  |  |  |  |  |  |  |  |  |  |  |
| --- | --- | --- | --- | --- | --- | --- | --- | --- | --- | --- | --- | --- | --- |
| Population Council (2019) - Pakistan | Situation Analysis of Reproductive Health of Adolescents and Youth in Pakistan | Yes | Yes | Yes | Somewhat | Somewhat | Somewhat | Yes | No | Somewhat | Yes | Valuable | High |
| Perera (2018) - Pakistan | 'When helpers hurt': Women's and midwives' stories of obstetric violence in state health institutions, Colombo district, Sri Lanka | Somewhat | Yes | Yes | Yes | Somewhat | No | Yes | Somewhat | Can't tell | Somewhat | Valuable | High |
| Hamid (2009) - Pakistan | Who am I? Where am I? Experiences of married young women in a slum in Islamabad, Pakistan | Yes | Yes | Somewhat | Somewhat | Yes | Yes | Yes | Yes | Somewhat | Somewhat | Valuable | High |
| \| Abbreviations: CASP; Critical Appraisal Skills Programme \| \| --- \| | | | | | | | | | | | | | |

| S2 Appendix. ii) Quality appraisal for medium relevance studies, as determined using the CASP quality appraisal tool and the relevance of the study to the review question, organised by country and year of publication | | | | | | | | | | | | | |
| --- | --- | --- | --- | --- | --- | --- | --- | --- | --- | --- | --- | --- | --- |
| Author (year) | Title | Was there a clear statement of the aims of the research? | Is a qualitative methodology appropriate? | Was the research design appropriate to address the aims of the research? | Was the recruitment strategy appropriate to the aims of the research? | Was the data collected in a way that addressed the research issue? | Has the relationship between researcher and participants been adequately considered? | Have ethical issues been taken into consideration? | Are the study’s theoretical underpinnings clear, consistent and conceptually coherent? | Was the data analysis sufficiently rigorous? | Is there a clear statement of findings? | How valuable is the research? | Considering the quality of the evidence and its relevance to the aims of this review, how relevant is this research to this review? |
| Kaartinen (2002) | Mother and child health care in Kabul, Afghanistan with focus on the mother: Women's own perspective | Yes | Yes | Yes | Yes | Somewhat | No | No | No | Can't tell | Somewhat | Somewhat valuable | Medium |
| Kwesiga (2021) | Barriers and enablers to reporting pregnancy and adverse pregnancy outcomes in population-based surveys: EN-INDEPTH study | Yes | Yes | Yes | Somewhat | Yes | Yes | Yes | No | Somewhat | Somewhat | Somewhat valuable | Medium |
| Ainul (2015) | Early marriage as a risk factor for mistimed pregnancy among married adolescents in Bangladesh | No | Yes | Somewhat | Can't tell | Somewhat | No | Yes | No | Can't tell | Somewhat | Somewhat valuable | Medium |
| HRW (2015) | Marry before your house is swept away: Child Marriage in Bangladesh | Yes | Somewhat | Yes | Can't tell | Can't tell | No | No | No | Can't tell | Yes | Valuable | Medium |
| Sikder (2011) | Accounts of severe acute obstetric complications in rural Bangladesh | Yes | Yes | Somewhat | Yes | Somewhat | Somewhat | Yes | No | Somewhat | Yes | Somewhat valuable | Medium |
| Naved (2005) | Factors associated with spousal physical violence against women in Bangladesh | Yes | Yes | Somewhat | Somewhat | Somewhat | No | Yes | No | Can't tell | Somewhat | Somewhat valuable | Medium |
| Bhuiya (2001) | Ordeal of women for induced abortion in a rural area of Bangladesh | Yes | Somewhat | Somewhat | Somewhat | Can't tell | No | No | No | Can't tell | Somewhat | Somewhat valuable | Medium |
| Khanna (2022) | Social and economic marginalisation and sexual and reproductive health and rights of urban poor young women: a qualitative study from Vadodara, Gujarat, India | Yes | Yes | Yes | Somewhat | Yes | Somewhat | Yes | No | Somewhat | Somewhat | Somewhat valuable | Medium |
| Sama (2019) | Interrogating Interruptions: Exploring Young Womens mental Health Issues | Yes | Yes | Yes | Yes | Yes | No | Yes | No | Yes | Yes | Valuable | Medium |
| Sansthan (2019) | What about us: A Study on the Situation of Young Married Girls | Yes | Yes | Yes | Yes | Can't tell | No | No | No | Can't tell | Yes | Somewhat valuable | Medium |
| Mukherjee (2018) | Dynamics of early marriage of girls in rural Uttar Pradesh: A study into the economic, social and human rights aspects; Chapter 7: Impact of Early Marriage on Women’s Autonomy, Sexual and Reproductive Health, and Mental Health | Yes | Somewhat | Yes | Can't tell | Can't tell | Somewhat | Yes | Somewhat | Can't tell | Yes | Valuable | Medium |
| Sahoo (2015) | Sanitation-related psychosocial stress: A grounded theory study of women across the life-course in Odisha, India | Yes | Yes | Yes | Somewhat | Yes | Somewhat | Yes | Yes | Yes | Yes | Valuable | Medium |
| Mitra (2015) | Child Marriage & Early Motherhood: Understandings from Lived Experiences of Young People | Somewhat | Can't tell | Yes | Yes | Can't tell | Somewhat | Yes | No | Can't tell | Somewhat | Valuable | Medium |
| Roberts (2012) | Grief and Women: Stillbirth in the Social Context of India | Yes | Yes | Yes | Yes | Yes | No | Yes | Somewhat | Yes | Yes | Somewhat valuable | Medium |
| HRW (2009) | No Tally of the Anguish: Accountability in Maternal Health Care in India | Yes | Yes | Yes | Yes | Can't tell | No | No | No | Can't tell | Yes | Valuable | Medium |
| Iyengar (2008) | Childbirth practices in rural Rajasthan, India: Implications for neonatal health and survival | Yes | Yes | Yes | Somewhat | Somewhat | Somewhat | Somewhat | No | Can't tell | Somewhat | Somewhat valuable |  |
| Mukhopadhyay (2004) | Mothers' perceptions and attitudes towards maternal morbidity in rural West Bengal: findings from focus group discussions | Yes | Yes | Somewhat | Yes | Somewhat | No | No | No | Yes | Somewhat | Valuable |  |
| Amin (2002) | The influence of gender on rural women's illness experiences and health-seeking strategies for gynaecological symptoms | Somewhat | Somewhat | Yes | Somewhat | Somewhat | No | Somewhat | No | Can't tell | Somewhat | Valuable |  |
| Morrison (2021) | Addressing anaemia in pregnancy in rural plains Nepal: A qualitative, formative study | Yes | Yes | Yes | Yes | Yes | Somewhat | Yes | Yes | Yes | Yes | Valuable |  |
| Rajbanshi (2021) | A qualitative study to explore the barriers for nonadherence to referral to hospital births by women with high-risk pregnancies in Nepal | Yes | Yes | Somewhat | Somewhat | Yes | Somewhat | Yes | Somewhat | Yes | Yes | Somewhat valuable |  |
| Sekine (2021) | Multilevel factors influencing contraceptive use and childbearing among adolescent girls in Bara district of Nepal: A qualitative study using the socioecological model | Yes | Yes | Yes | Yes | Yes | Somewhat | Yes | Yes | Yes | Yes | Valuable | Medium |
| Bhandari (2016) | Perception and Perceived Experiences about Prevention and Consequences of Teenage Pregnancy and Childbirth among Teenage Mothers: A Qualitative Study | Yes | Yes | Somewhat | Can't tell | Can't tell | No | Yes | No | Somewhat | Somewhat | Somewhat valuable | Medium |
| Deuba (2016) | Experience of intimate partner violence among young pregnant women in urban slums of Kathmandu Valley, Nepal: A qualitative study | Yes | Yes | Somewhat | Somewhat | Yes | Somewhat | Yes | No | Somewhat | Somewhat | Valuable | Medium |
| HRW (2016) | "Our Time to Sing and Play": Child Marriage in Nepal | No | Can't tell | Yes | Can't tell | Can't tell | No | No | No | Can't tell | Yes | Valuable | Medium |
| Raj Baral (2016) | The uptake of skilled birth attendants' services in rural Nepal: A qualitative study | Yes | Yes | Yes | Yes | Yes | No | Yes | No | No | Somewhat | Valuable | Medium |
| Kaphle (2013) | Childbirth traditions and cultural perceptions of safety in nepal: Critical spaces to ensure the survival of mothers and newborns in remote mountain villages | Yes | Yes | Yes | Somewhat | Somewhat | Somewhat | Yes | Yes | No | Yes | Valuable | Medium |
| Simkhada (2011) | Antenatal care uptake in Nepal: barriers and opportunities | Yes | Yes | Yes | Yes | Yes | Yes | Yes | No | Yes | Yes | Valuable | Medium |
| Brunson (2010) | Confronting maternal mortality, controlling birth in Nepal: the gendered politics of receiving biomedical care at birth3 | Yes | Yes | Yes | Somewhat | Yes | Yes | Somewhat | Yes | Can't tell | Somewhat | Valuable | Medium |
| Matsuyama (2002) | Health -seeking behavior of women and their families during pregnancy, delivery and postpartum period in Nepal | Yes | Yes | Yes | Yes | Yes | No | Yes | Somewhat | Can't tell | Somewhat | Valuable | Medium |
| Gittelsohn (1997) | Cultural factors, caloric intake and micronutrient sufficiency in rural Nepali households | Yes | Yes | Can't tell | Somewhat | Somewhat | No | No | Can't tell | Can't tell | Somewhat | Somewhat valuable | Medium |
| Sultana (2022) | A phenomenological Analysis of Rural Women’s Childbirth Preferences | Yes | Yes | Somewhat | Can't tell | Can't tell | Somewhat | Yes | No | No | Yes | Somewhat valuable | Medium |
| Omer (2021) | The influence of social and cultural practices on maternal mortality: a qualitative study from South Punjab, Pakistan | Yes | Yes | Yes | Somewhat | Yes | No | Yes | Somewhat | Somewhat | Somewhat | Valuable | Medium |
| Yes I Do (2018) | Gaining insight into the magnitude of and factors influencing child marriage and teenage pregnancy and their consequences in Pakistan | Yes | Yes | Yes | Yes | Can't tell | Somewhat | Yes | Yes | Somewhat | Yes | Valuable | Medium |
| Nasrullah (2014) | Circumstances leading to intimate partner violence against women married as children: A qualitative study in Urban Slums of Lahore, Pakistan | Yes | Somewhat | Yes | Somewhat | Yes | Somewhat | Yes | No | Yes | Somewhat | Somewhat valuable | Medium |
| Rizvi (2014) | Gender: Shaping personality, lives and health of women in Pakistan | Yes | Yes | Somewhat | Yes | Somewhat | No | No | Somewhat | Somewhat | Yes | Valuable | Medium |
| Mumtaz (2009) | Understanding gendered influences on women's reproductive health in Pakistan: Moving beyond the autonomy paradigm | Yes | Somewhat | Yes | Yes | Somewhat | No | Somewhat | Yes | Somewhat | Yes | Valuable | Medium |
| Hussain (2008) | Women's perceptions and experiences of sexual violence in marital relationships and its effect on reproductive health | Yes | Yes | Yes | Yes | Yes | No | No | No | Somewhat | Yes | Valuable | Medium |
| Mumtaz (2007) | Gender, pregnancy and the uptake of antenatal care services in Pakistan | Somewhat | Yes | Yes | Yes | Somewhat | Yes | No | Somewhat | Yes | Somewhat | Somewhat valuable | Medium |
| Winkvist (2000) | God should give daughters to rich families only: Attitudes towards childbearing among low-income women in Punjab, Pakistan | Somewhat | Yes | Yes | Somewhat | Yes | Somewhat | Somewhat | No | Somewhat | Yes | Somewhat valuable | Medium |
| Abbreviations: CASP; Critical Appraisal Skills Programme, HRW; Human Rights Watch, TISS; Tata Institute of Social Sciences | | | | | | | | | | | | | |

| S2 Appendix. iii) Quality appraisal for low relevance studies, as determined using the CASP quality appraisal tool and the relevance of the study to the review question, organised by country and year of publication | | | | | | | | | | | | | |
| --- | --- | --- | --- | --- | --- | --- | --- | --- | --- | --- | --- | --- | --- |
| Author (year) | Title | Was there a clear statement of the aims of the research? | Is a qualitative methodology appropriate? | Was the research design appropriate to address the aims of the research? | Was the recruitment strategy appropriate to the aims of the research? | Was the data collected in a way that addressed the research issue? | Has the relationship between researcher and participants been adequately considered? | Have ethical issues been taken into consideration? | Are the study’s theoretical underpinnings clear, consistent and conceptually coherent? | Was the data analysis sufficiently rigorous? | Is there a clear statement of findings? | How valuable is the research? | Considering the quality of the evidence and its relevance to the aims of this review, how relevant is this research to this review? |
| Christou (2020) | Understanding pathways leading to stillbirth: The role of care-seeking and care received during pregnancy and childbirth in Kabul province, Afghanistan | Yes | Yes | Yes | Yes | Yes | No | Yes | Yes | Yes | Yes | Valuable | Low |
| Arnold (2019) | Villains or victims? An ethnography of Afghan maternity staff and the challenge of high quality respectful care | Yes | Yes | Yes | Somewhat | Yes | Somewhat | Yes | No | Yes | Yes | Valuable | Low |
| Raj (2014) | Multisectorial Afghan Perspectives on Girl Child Marriage: Foundations for Change Do Exist in Afghanistan | Yes | Yes | Yes | Somewhat | Somewhat | No | Yes | No | No | Yes | Valuable | Low |
| Biswas (2020) | Exploring the perceptions, practices and challenges to maternal and newborn health care among the underprivileged teagarden community in Bangladesh: a qualitative study | Yes | Yes | Yes | Somewhat | Yes | No | Yes | No | Somewhat | Somewhat | Valuable | Low |
| Barua (2018) | Tuberculosis and the sexual and reproductive lives of women in Bangladesh | Yes | Yes | Yes | Yes | Yes | No | Yes | No | Somewhat | Yes | Valuable | Low |
| Alam (2015) | How can formative research inform the design of an iron-folic acid supplementation intervention starting in first trimester of pregnancy in Bangladesh? | Yes | Yes | Yes | Somewhat | Can't tell | No | Yes | No | Can't tell | Somewhat | Valuable | Low |
| Rasweth (2022) | The unspoken plight of married adolescent girls in rural Tamil Nadu: Narrative summary on unmet sexual and reproductive health needs and barriers. | Yes | Yes | Yes | Can't tell | Somewhat | No | Yes | No | Somewhat | No | Somewhat valuable | Low |
| Mayra (2022) | Why do some health care providers disrespect and abuse women during childbirth in India? | Yes | Yes | Somewhat | Yes | Somewhat | Somewhat | Yes | No | Somewhat | Somewhat | Somewhat valuable | Low |
| Bhatia (2021) | Perception and health seeking behaviour of people regarding anaemia: An experience from Odisha in Eastern India | Yes | Yes | Somewhat | Can't tell | Can't tell | No | Yes | No | Can't tell | No | Somewhat valuable | Low |
| Doke (2021) | Meager Perception of Preconception Care Among Women Desiring Pregnancy in Rural Areas: A Qualitative Study Using Focus Group Discussions | Yes | Yes | Somewhat | Somewhat | Yes | No | Yes | No | Somewhat | Somewhat | Somewhat valuable | Low |
| Gopichandran (2018) | Psycho-social impact of stillbirths on women and their families in Tamil Nadu, India - a qualitative study | Yes | Yes | Yes | Somewhat | Somewhat | Yes | Yes | Somewhat | Yes | Yes | Somewhat valuable | Low |
| Sarkar (2018) | Factors influencing the place of delivery in rural Meghalaya, India: a qualitative study | Yes | Yes | Yes | Can't tell | Somewhat | No | No | No | No | No | Somewhat valuable | Low |
| Seth (2018) | Social determinants of child marriage in rural india | No | Can't tell | Somewhat | Can't tell | Can't tell | Somewhat | No | No | Can't tell | Somewhat | Not valuable | Low |
| Bhattacharyya (2017) | Perceptions of accredited social health activists regarding teen age pregnancy: A qualitative study in a rural area of West Bengal, India | Yes | Yes | Somewhat | Yes | Yes | No | Somewhat | No | Somewhat | Somewhat | Somewhat valuable | Low |
| Vlassoff (2017) | Can conditional cash transfers promote delayed childbearing? Evidence from the 'Second Honeymoon Package' in rural Maharashtra, India | Yes | Somewhat | Somewhat | Yes | Can't tell | No | No | No | Can't tell | No | Somewhat valuable | Low |
| Ramakrishnan (2012) | Public health interventions, barriers, and opportunities for improving maternal nutrition in India | Somewhat | Somewhat | Yes | Yes | Yes | No | Yes | No | Can't tell | Somewhat | Valuable | Low |
| Awasthi (2006) | Danger signs of neonatal illnesses: Perceptions of caregivers and health workers in northern India | Yes | Yes | Yes | Somewhat | Somewhat | No | Yes | No | No | Somewhat | Valuable | Low |
| Chorghade (2006) | Why are rural Indian women so thin? Findings from a village in Maharashtra | Yes | Yes | Somewhat | Somewhat | Somewhat | No | No | No | Yes | Yes | Valuable | Low |
| FRHS (n.d.) | Role of Mothers-in-Law in Young Women's Reproductive Health: Evidence from Intervention Research in Rural Maharashtra, India | Somewhat | Somewhat | No | Can't tell | Can't tell | No | No | No | No | Somewhat | Not valuable | Low |
| Rajbanshi (2021) | Perceptions of good-quality antenatal care and birthing services among postpartum women in Nepal | Yes | Yes | No | Somewhat | Yes | Somewhat | Yes | No | Somewhat | Yes | Somewhat valuable | Low |
| Shah (2018) | Barriers and facilitators to institutional delivery in rural areas of Chitwan district, Nepal: A qualitative study | Yes | Yes | Yes | Somewhat | Yes | Somewhat | Yes | No | Yes | Yes | Valuable | Low |
| Care (2016) | The cultural context of child marriage in Nepal and Bangladesh | Yes | Yes | Yes | Can't tell | Can't tell | No | No | Somewhat | Yes | Somewhat | Valuable | Low |
| Mahato (2016) | Causes and Consequences of Child Marriage: A Perspective | Yes | Somewhat | Somewhat | Somewhat | Can't tell | No | No | No | Can't tell | Somewhat | Somewhat valuable | Low |
| Ali (2021) | Perceptions of women, their husbands and healthcare providers about anemia in rural Pakistan: Findings from a qualitative exploratory study | Yes | Yes | Yes | Yes | Yes | No | Yes | No | Somewhat | Somewhat | Somewhat valuable | Low |
| Ehsan (2021) | Domestic violence against pregnant women and its effects on their reproductive health | Yes | Yes | No | Can't tell | Can't tell | No | Somewhat | No | Can't tell | No | Somewhat valuable | Low |
| Wyatt (2021) | Predictors and occurrence of antenatal depressive symptoms in Galle, Sri Lanka: a mixed-methods cross-sectional study | Yes | Yes | Somewhat | Somewhat | Yes | Somewhat | Yes | No | No | Yes | Somewhat valuable | Low |
| Abbreviations: FRHS; Foundation for Reproductive Health Services; | | | | | | | | | | | | | |
