# Supplementary material for: “Can’t live willingly”: A thematic synthesis of qualitative evidence exploring how early marriage and early pregnancy affect experiences of pregnancy in South Asia
Source: PLOS Glob Public Health. 2023 Oct 23;3(10):e0002279. doi: 10.1371/journal.pgph.0002279 (PMC10593245; doi:10.1371/journal.pgph.0002279)
Supplement: S3 Appendix — (DOCX) [file pgph.0002279.s003.docx]

| S3 Appendix. i) Summary of medium relevance studies (arranged by country and descending year) | | | | | |
| --- | --- | --- | --- | --- | --- |
| Author | Title | Country and region of participants | Participants | Methods | Topic |
| Kaartinen (2002) | Mother and child health care in Kabul, Afghanistan with focus on the mother: Women's own perspective | Afghanistan (Qala-e-Shada, Kabul) | 100 SSI: Women who delivered ≤10 years, TBAs, teachers | SSI  Distinguishing categories in data, no theoretical underpinnings | Health care seeking behaviour among women with childbirth experience |
| Kwesiga (2021) | Barriers and enablers to reporting pregnancy and adverse pregnancy outcomes in population-based surveys: EN-INDEPTH study | Bangladesh (Matlab; also in Guinea Bissau, Ethiopia, Uganda and Ghana) | unknown # FGD: 28 EN-INDEPTH participants, 23 EN-INDEPTH interviewers | FGD Thematic analysis using interpretive paradigm and phenomenology | Individual, community, cultural and interview factors affecting participation |
| Ainul (2015) | Early marriage as a risk factor for mistimed pregnancy among married adolescents in Bangladesh | Bangladesh (unclear which district) | 9 case studies: Married and unmarried adolescents | IDI for case histories No theoretical underpinnings | Sexual and reproductive health, pregnancy history and relationship dynamics |
| HRW (2015) | Marry before your house is swept away: Child Marriage in Bangladesh | Bangladesh (Noakhali, Laxmipur, Khulna, Sirajganj, Gaibandha and Dhaka) | 114  59 women and girls married <18 years in last 5 years, their families 24 local officials, teachers, NGO workers, and representatives of international organizations | IDI and KII No analysis method, no theoretical underpinnings | Vulnerability to early marriage in areas affected by natural disasters |
| Sikder (2011) | Accounts of severe acute obstetric complications in rural Bangladesh | Bangladesh (Gaibandha) | 40 women: 9 with experience of haemorrhage, 8 of obstructed labour, 7 of eclampsia, 7 of sepsis, 9 of induced abortion | SSI Thematic analysis to build conceptual model | Experiences of severe obstetric complications |
| Naved (2005) | Factors associated with spousal physical violence against women in Bangladesh | Bangladesh (unclear which district; rural and urban) | 28 women who had experienced abuse | IDI No analysis method, as part of a mixed-methods study, conceptual framework provided | Illustrate personal determinants of violence and personalise the violence |
| Bhuiya (2001) | Ordeal of women for induced abortion in a rural area of Bangladesh | Bangladesh (Matlab and Daudkandi, Chandpur) | 21 IDI: Women with induced abortion or family members if women deceased | IDI Case studies, no theoretical underpinnings | Process and management practices of abortion |
| Khanna (2022) | Social and economic marginalisation and sexual and reproductive health and rights of urban poor young women: a qualitative study from Vadodara, Gujarat, India | India (Gujurat) | 16 IDI: 14 young women, 2 front-line health workers 2 FGD (8/group): 16 young women | IDI and FGD Thematic analysis, no theoretical underpinnings | The sexual and reproductive lives of young women, from a sexual and reproductive health and rights perspective |
| Sama (2019) | Interrogating Interruptions: Exploring Young Women's mental Health Issues | India (Rajasthan and Uttar Pradesh) | 42 IDI: young married and unmarried women (18-25) 10 FGD (UK#/group): young (18-25) and older (25-35) married and unmarried women  39 KII: Local organisation representatives, teachers, health workers (doctors, TBAs, psychiatrists), caregivers of participants with mental illness | IDI, FGD and KII Thematic analysis, no theoretical underpinnings |  |
| Sansthan (2019) | What about us: A Study on the Situation of Young Married Girls | India (Udaipur, Rajasthan State) | 218 young women 200 older women 50 young men 13 community members | SSI and IDI No analysis method, no theoretical underpinnings | Personal motivation and commitment to work on early marriage |
| Mukherjee (2018) | Dynamics of early marriage of girls in rural Uttar Pradesh: A study into the economic, social and human rights aspects; Chapter 7: Impact of Early Marriage on Women’s Autonomy, Sexual and Reproductive Health, and Mental Health | India (Shravasti, Uttar Pradesh) | unknown # case studies: married women unknown # FGD: community members | Case studies and IDI No analysis method or theoretical underpinnings | Consequences of early marriage |
| Sahoo (2015) | Sanitation-related psychosocial stress: A grounded theory study of women across the life-course in Odisha, India | India (urban slums, rural villages and tribal villages, Odisha) | 56 unmarried adolescent girls, newly married women, pregnant women and not-pregnant married women who married >2 years | IDI Grounded theory within life-course perspective | Experiences of and adaptation to sanitation routines |
| Mitra (2015) | Child Marriage & Early Motherhood: Understandings from Lived Experiences of Young People | India (Andhra Pradesh, Telangana, Assam, Bihar, Madhya Pradesh, Meghalaya, Rajasthan and West Bengal) | 57 life stories: 42 girls/women, 15 boys/men 40 FGD (UK#/group): 316 community members | Life histories, FGD No analysis method or theoretical underpinnings | Experiences of and rationale for early marriage |
| Roberts (2012) | Grief and Women: Stillbirth in the Social Context of India | India (rural central India) | 31 women with history of stillbirth (15 personal history, 16 observers)  2 husbands of wives who had stillbirth  17 KII: hospital staff, health care professionals, patients and family members, and TBA unknown # FGD: 6 with no experience of stillbirth, 10 older women (3 with experience of stillbirth) | KII and FGD Grounded theory | Experience, knowledge and beliefs on stillbirths |
| HRW (2009) | No Tally of the Anguish: Accountability in Maternal Health Care in India | India (Rae Bareilly, Unnao, Chitrakoot, Lucknow and Barabanki, Uttar Pradesh; New Delhi and Tamil Nadu) | Interviews and FGD: 55 public health specialists Uttar Pradesh 56 women and men from villages  34 health staff (government health facilities, ANM, ASHAs, female community health aides, anganwadi workers and TBA) 45 officials 7 private doctors 30 journalists/NGO staff New Delhi: 11 officials Tamil Nadu: 4 former and 4 present government officials | FGD and IDI No analysis method, guided by human rights framework | Accountability in preventable maternal mortality and morbidity |
| Iyengar (2008) | Childbirth practices in rural Rajasthan, India: Implications for neonatal health and survival | India (rural Udaipur) | 10 FGD (8-12/group): Mothers, grandmothers and TBA 18 case studies: Recently delivered women 8 observations of delivery 39 KII: Mothers, grandmothers and TBAs | FGD, case studies, observations, KII Iterative coding, no theoretical underpinnings | Practices during labour and delivery |
| Mukhopadhyay (2004) | Mothers' perceptions and attitudes towards maternal morbidity in rural West Bengal: findings from focus group discussions | India (Malda, Uttar Dinajpur and Dakshin Dinajpur, West Bengal) | 3 FGD (8-10/group): young women of reproductive age, older women (≥45 years), TBA and MPHW | FGD Organised into categories and sub-topics, no theoretical underpinnings | Perception and attitudes towards maternal (obstetric) morbidity |
| Amin (2002) | The influence of gender on rural women's illness experiences and health-seeking strategies for gynaecological symptoms | India (Panchmahals, Gujarat) | 9 KII: NGO staff members and community leaders 9 FGD (7-10/group): 1 with barefoot gynaecologists, 1 with TBA, and 7 with women from SARTHI groups 18 illness narratives | KII, FGD and illness narratives Analysis guided by gender inequality | Reproductive health and illnesses |
| Morrison (2021) | Addressing anaemia in pregnancy in rural plains Nepal: A qualitative, formative study | Nepal (Kapilvastu) | 24 SSI: 16 women with children ≤6 months, 4 nurses, 4 key informants (NGO staff, FCHVs and religious leaders) 3FGD (unknown #/group): 20 fathers 3FGD (unknown #/group): 19 MI | SSI and FGD Descriptive content analysis, socio-ecological approach | Anaemia and nutrition |
| Rajbanshi (2021) | A qualitative study to explore the barriers for nonadherence to referral to hospital births by women with high-risk pregnancies in Nepal | Nepal (Morang) | 14 postpartum women with high-risk factors | SSI Thematic analysis and phenomenological approach | Perceptions of good-quality ANC and birthing services |
| Sekine (2021) | Multilevel factors influencing contraceptive use and childbearing among adolescent girls in Bara district of Nepal: A qualitative study using the socioecological model | Nepal (rural and urban Bara) | 60 IDI: 20 adolescent, 20 husbands, 20 MIL 10 KII: 4 HC providers, 3 coordinators, 3 FCHV | KII and IDI Content analysis organised according to socioecological model | Factors influencing contraceptive use and childbearing among adolescent girls |
| Bhandari (2016) | Perception and Perceived Experiences about Prevention and Consequences of Teenage winPregnancy and Childbirth among Teenage Mothers: A Qualitative Study | Nepal (Kathmandu and Patan) | 24 IDI: Teenage mothers unknown # FGD: 24 care takers of teenage mothers | FGF and IDI Phenomenological approach, no theoretical underpinnings | Perception and experiences among teenage mothers |
| Deuba (2016) | Experience of intimate partner violence among young pregnant women in urban slums of Kathmandu Valley, Nepal: A qualitative study | Nepal (Urban slums, Kathmandu valley) | 20 young pregnant women | IDI Content analysis, no theoretical underpinnings | Perceptions and experiences of IPV |
| HRW (2016) | "Our Time to Sing and Play": Child Marriage in Nepal | Nepal (Banke, Bara, Chitwan, Gorkha, Kailali, Kathmandu, Morang, Nawalparasi, Rupandehi, Saptari, Sarlahi, Sindhupalchuk, Siraha and Sunsari) | 149 IDI: 38 married children, 66 adults married as children (most women, 8 men), 28 KII (educators, health workers, police officers, community leaders and NGO experts) | IDI and KII No theoretical underpinnings | How early marriage has affected the lives of young girls |
| Raj Baral (2016) | The uptake of skilled birth attendants' services in rural Nepal: A qualitative study | Nepal (Western Development Region) | 24 women who had given birth ≤3 years 8 relatives | SSI Thematic analysis, no theoretical underpinnings | Uptake of SBA services during childbirth |
| Kaphle (2013) | Childbirth traditions and cultural perceptions of safety in Nepal: Critical spaces to ensure the survival of mothers and newborns in remote mountain villages | Nepal (Mugu) | 46 IDI: 25 pregnant or postnatal women, 5 husbands, 5 MIL, 1 FIL, 5 service providers and 5 local stakeholders | IDI Thematic analysis, guided by social constructionist and critical feminist theories | Interplay between traditional beliefs and pregnancy and childbirth practices |
| Simkhada (2011) | Antenatal care uptake in Nepal: barriers and opportunities | Nepal (Daksindkali and Caimale, semi-urban areas Kathmandu) | 15 women who had used ANC, 5 husbands, 6 MIL 15 women had NOT used ANC, 5 husbands, 4 MIL | IDI Grounded theory | Opportunities and barriers in ANC uptake |
| Brunson (2010) | Confronting maternal mortality, controlling birth in Nepal: the gendered politics of receiving biomedical care at birth | Nepal (Semi-urban village, Kathmandu Valley) | 30 case studies: Women of reproductive age | Ethnography; SSI and observations Concept-driven coding, guided by birth preparedness (biomedical/risk) framework | Marriage, work, pregnancy, birth and postpartum experiences, and the role of women |
| Matsuyama (2002) | Health -seeking behaviour of women and their families during pregnancy, delivery and postpartum period in Nepal | Nepal (Kavrepalanchowk) | 28 informants: women with small children, MIL, husbands, Dhami-jankri, TBA, health centre staff, and drug store keepers 74 case histories: women with small children or family members Free-listing and pile sort: 20 women, 20 MIL, 20 husbands, 10 TBA and 10 Dhami-jankri 64 EDM: women with small children | KII, case histories, free-listing, pile sorting, EDM Ethnographic, analysed using three delays framework. Developed theoretical framework for the association between women's education and their own health | How socio-cultural factors affect decision about attending antenatal care |
| Gittelsohn (1997) | Cultural factors, caloric intake and micronutrient sufficiency in rural Nepali households | Nepal (Western Hills) | unknown # KII: Households unknown # FGD (2-6/group): Individuals from households | KII and FGD No analysis method, conceptual framework presented | Intrahousehold food allocation |
| Sultana (2022) | A Phenomenological Analysis of Rural Women’s Childbirth Preferences | Pakistan (Punjab) | 60 IDI: women of reproductive age unknown # FGD (unknown #/group): Community members and service providers such as traditional birth attendants, lady health visitors, spiritual healers and alternative providers 'Input' from unknown # husbands, doctors, nurses, and parallel medical service sectors such as herbalists, quacks, spiritual healers, and other village informants | IDI and FGD Phenomenological analysis, use of narratives | Psychological and socio-cultural dimensions regarding women’s reproductive processes |
| Omer (2021) | The influence of social and cultural practices on maternal mortality: a qualitative study from South Punjab, Pakistan | Pakistan (Dera Ghazi Khan, South Punjab) | 60 KII: gynaecologists  4 FGD (unknown #/group): LHW 10 case studies: family of deceased mothers | KII, FGD and case studies Thematic analysis, three delays model | Socio-cultural factors contributing to a delay in seeking maternal healthcare |
| Yes I Do (2018) | Gaining insight into the magnitude of and factors influencing child marriage and teenage pregnancy and their consequences in Pakistan | Pakistan (Umerkot and Sanghar) | 10 FGD (7-9/group): 88 women  8 IDI: 3 education professionals, 2 health professionals, 2 community leaders, 1 religious leaders 3 KII: policy makers | FGD, IDI, KII Content analysis, situated within their theory of change | Experience and opinions related to SRHR and related policies |
| Nasrullah (2014) | Circumstances leading to intimate partner violence against women married as children: A qualitative study in Urban Slums of Lahore, Pakistan | Pakistan (Lahore) | 19 women married <18 years for ≥5 years | IDI Thematic analysis, no theoretical underpinnings | Types and circumstances of IPV |
| Rizvi (2014) | Gender: Shaping personality, lives and health of women in Pakistan | Pakistan (slum in Karachi) | 250 women living in slums | FGD Content analysis, no theoretical underpinnings | Gender roles related to reproductive health |
| Mumtaz (2009) | Understanding gendered influences on women's reproductive health in Pakistan: Moving beyond the autonomy paradigm | Pakistan (Punjab) | 171 observations: community people 35 IDI: 15 women, 15 husbands, 5 MIL 6 FGD (6-10/group): women (4) and men (2) 5 case studies: women, husbands and family members | Social mapping, observations, IDI, FGD and case studies Ethnographic and content analysis | Sociocultural construction of gender and implications for ANC utilisation |
| Hussain (2008) | Women's perceptions and experiences of sexual violence in marital relationships and its effect on reproductive health | Pakistan (Karachi) | KII: 8 older women 3 FGD (5-12/group): 24 women aged 20-70 years IDI: 10 women currently experiencing GBV | KII, FGD, IDI Thematic analysis, no theoretical underpinnings | Sexual violence and impact on reproductive health |
| Mumtaz (2007) | Gender, pregnancy and the uptake of antenatal care services in Pakistan | Pakistan (Pind, Punjab) | 171 observations: community people 35 IDI: 15 women, 15 husbands, 5 MIL 6 FGD (6-10/group): women (4) and men (2) 5 case studies: women, husbands and family members | Social mapping, observations, IDI, FGD and case studies Content analysis and grounded theory | Sociocultural construction of gender and implications for ANC utilisation |
| Winkvist (2000) | God should give daughters to rich families only: Attitudes towards childbearing among low-income women in Punjab, Pakistan | Pakistan (urban and rural Lahore) | 42 women: 24 village, 18 city 8 MIL 6 health care providers: 2 TBA, 2 medical doctor, 1 traditional healer, 1 family planning representative | IDI and FGD Grounded theory | Women's perceptions and experiences of bearing sons and daughters |
| Abbreviations: ANC; antenatal care, ANM; auxiliary nurse midwife, CCT; conditional cash transfers, CHW; community health worker, DPHO; district public health officer, EDM; ethnographic decision models, FCHV; female community health volunteer, FGD; focus group discussion, GBV; gender-based violence, HRW; Human Rights Watch, IDI; in-depth interview, IPV; intimate partner violence, IUGR; intra-uterine growth restriction KII; key-informant interview, LWH; lady health worker, MIL; mother-in-law, MPHW; multi-purpose health worker, n.d.; no date, NGO; non-governmental organisation; PHM; public health midwife, SBA; skilled birth attendant, SRHR; sexual and reproductive health rights, SSI; semi-structured interviews, TBA: traditional birth attendant, y/o; year old, yr; year, unknown #; unknown number | | | | | |

| S3 Appendix. ii) Summary of low relevance studies (arranged by country and descending year) | | | | | |
| --- | --- | --- | --- | --- | --- |
| Author | Title | Country and region of participants | Participants | Methods | Outcome/area of focus |
| Christou (2020) | Understanding pathways leading to stillbirth: The role of care-seeking and care received during pregnancy and childbirth in Kabul province, Afghanistan | Afghanistan (Kabul) | 55 IDI: Mothers and fathers with recent experience of stillbirth, female community elders, CHWs, healthcare providers at maternity hospitals, and government health officials | IDI Thematic analysis, guided by three delays framework | Experiences, perceptions and practices around stillbirth |
| Arnold (2019) | Villains or victims? An ethnography of Afghan maternity staff and the challenge of high quality respectful care | Afghanistan (Kabul) | 23 SSI: Hospital staff 16 SSI: Kabul women 41 KII: 19 Afghans and 22 non-Afghans | SSI and KII Thematic analysis, no theoretical underpinnings | Experiences, perspectives and motivations of maternity staff |
| Raj (2014) | Multisectoral Afghan Perspectives on Girl Child Marriage: Foundations for Change Do Exist in Afghanistan | Afghanistan (Kabul, Jalalabad, and Mazar) | 112 people associated with NGOs, government institutions, police departments, schools, and mosques | KII, FGD Grounded theory | Knowledge and attitudes of child and forced marriage |
| Biswas (2020) | Exploring the perceptions, practices and challenges to maternal and newborn health care among the underprivileged teagarden community in Bangladesh: a qualitative study | Bangladesh (Moulvibazar) | 6 FGD (8-10/group): 54 males and females from teagardens 12 IDI: Health facility staff in teagardens | FGD and IDI Content analysis, no theoretical underpinnings | Service delivery in maternal newborn health |
| Barua (2018) | Tuberculosis and the sexual and reproductive lives of women in Bangladesh | Bangladesh (slum in Dhaka) | 15 IDI: 9 women, 4 husbands, 2 MIL | IDI Narrative analysis, no theoretical underpinnings | Experience of married women with TB |
| Alam (2015) | How can formative research inform the design of an iron-folic acid supplementation intervention starting in first trimester of pregnancy in Bangladesh? | Bangladesh (villages in Mymensing and Netrokona districts) | 66 IDI: 35 pregnant women, 20 older women, 11 fathers 20 KII: CHW, program managers, informal care providers, gynaecologists, traditional birth attendants 3 FGD (unknown #/group): 2 with Frontline health workers, 1 with female college students | IDI, KII, FGD No theoretical underpinnings# | Cultural norms affecting IFA supplementation during pregnancy |
| Rasweth (2022) | The unspoken plight of married adolescent girls in rural Tamil Nadu: Narrative summary on unmet sexual and reproductive health needs and barriers | India (Tamil Nadu) | 14 IDI: Married women 14 FGD (unknown #/group): Their spouses, mothers-in-law, and fathers-in-law | IDI and FGD Narrative thematic analysis | The sexual and reproductive needs of adolescent married girls, including barriers and enablers |
| Mayra (2022) | Why do some health care providers disrespect and abuse women during childbirth in India? | India (Bihar, Rajasthan, Odisha, Madhya Pradesh and West Bengal) | 34 nursing and midwifery leaders | IDI Thematic analysis, no theoretical underpinnings | Nurse-midwives perspective on the reasons behind mistreatment of women during childbirth |
| Bhatia (2021) | Perception and health seeking behaviour of people regarding anaemia: An experience from Odisha in Eastern India | India (Jagatsinghpur, Bhadrak, Kalahandi and Keonjhar, Odisha) | FGD (unknown #/group): 34 beneficiaries (e.g. Block Programme Manager, ASHAs, teachers) | FGD Descriptive analysis, no theoretical underpinnings | Perception regarding anaemia |
| Doke (2021) | Meagre Perception of Preconception Care Among Women Desiring Pregnancy in Rural Areas: A Qualitative Study Using Focus Group Discussions | India (Nashik, Maharashtra) | FGD (6-12/group): 76 Reproductive age women with desire to have child in next year | FGD Content analysis, no theoretical underpinnings | Pre-conception care behaviour and services |
| Gopichandran (2018) | Psycho-social impact of stillbirths on women and their families in Tamil Nadu, India - a qualitative study | India (Tamil Nadu) | 8 IDI: Women with experience of stillbirth in past 1 year | IDI Thematic analysis to develop psychosocial conceptual model | Social and emotional impact of stillbirths |
| Sarkar (2018) | Factors influencing the place of delivery in rural Meghalaya, India: a qualitative study | India (Meghalaya) | 58 pregnant women, elderly women, ASHA, ANM and SBA | FGD and IDI Deductive thematic content analysis using a priori themes, no theoretical underpinnings | Reasons for home or health facility for delivery |
| Seth (2018) | Social determinants of child marriage in rural India | India (rural Mewat) | 2 families | Case studies based on interviews and observation No theoretical underpinnings | Social determinants of early marriage |
| Bhattacharyya (2017) | Perceptions of accredited social health activists regarding teen age pregnancy: A qualitative study in a rural area of West Bengal, India | India (West Bengal) | 1 FGD: 10 ASHAs | FGD Thematic analysis, no theoretical underpinnings | Perception of teenage pregnancy and preventative methods |
| Vlassoff (2017) | Can conditional cash transfers promote delayed childbearing? Evidence from the 'Second Honeymoon Package' in rural Maharashtra, India | India (Satar, Maharashtra) | 6 officials 5 beneficiaries  1 FGD: 9 health workers | Interviews and FGD No analysis method or theoretical underpinnings | Implementation of and experience of CCTs to newly married couples to postpone their first birth |
| Ramakrishnan (2012) | Public health interventions, barriers, and opportunities for improving maternal nutrition in India | India (Uttar Pradesh and Tamil Nadu) | Tamil Nadu 17 KII: National and state level policy and decision makers 15 FGD: CHW and women of reproductive age 6 IDI: ANM, local doctors, ASHA and local leaders  Uttar Pradesh 14 KII: National and state level policy and decision makers 19 FGD: CHW and women of reproductive age 9 IDI: ANM, local doctors, ASHA and local leaders | KII, IDI, FGD Deductive coding across a-priori themes: anaemia, IUGR and maternal undernutrition | Explore existing platforms for, and gaps and barriers to, maternal nutrition intervention |
| Awasthi (2006) | Danger signs of neonatal illnesses: Perceptions of caregivers and health workers in northern India | India (Lucknow, Uttar Pradesh) | 23 IDI: Caregivers 7 FGD: 3 with mothers, 2 with grandmothers/other female relatives), 1 with ANM, 1 with TBA 12 KII with providers: 4 doctors, 4 other health workers, 2 TBA, 2 volunteer health workers. | IDI, FGD and KII Analysed for keywords and phrases, no theoretical underpinnings | Household practices that affect neonatal health |
| Chorghade (2006) | Why are rural Indian women so thin? Findings from a village in Maharashtra | India (Maharashtra) | 12 FGD (6-8/group): Young mothers, young fathers, grandmothers and grandfathers | FGD Thematic analysis, no theoretical underpinnings | Explanations for and perceptions of thinness of young women |
| FRHS (n.d.) | Role of Mothers-in-Law in Young Women's Reproductive Health: Evidence from Intervention Research in Rural Maharashtra, India | India (rural Maharashtra) | 150 IDI: 75 young married women and their MIL | IDI No analysis method, no theoretical underpinnings | Attitudes towards and decision making in reproductive health |
| Rajbanshi (2021) | Perceptions of good-quality antenatal care and birthing services among postpartum women in Nepal | Nepal (Morang) | 14 pregnant adolescents or within 42 days of birth, with high-risk pregnancy who did not comply with hospital referrals | IDI  *Thematic analysis, within behaviour change theory* | Reasons for non-adherence to hospital referral |
| Shah (2018) | Barriers and facilitators to institutional delivery in rural areas of Chitwan district, Nepal: A qualitative study | Nepal (Chitwan) | 10 FGD (UK#/group): 4 with women who had home delivery, 4 with women who had institutional delivery, 2 with MIL 12: IDI: 2 husbands, 2 TBAs, 2 FCHVs, 2 service providers health post, 2 ANM, 1 DPHO, 1 safe motherhood focal person | FGD and IDI Thematic analysis, no theoretical underpinnings | Socio-cultural and health service-related barriers to and facilitators of institutional delivery |
| Care (2016) | The cultural context of child marriage in Nepal and Bangladesh | Nepal (Rupandehi and Kapilvastu) and Bangladesh (Sunamganj) | Bangladesh: unknown # social mapping: Men and women 16-20 timelines: Fathers and mothers unknown # seasonal calendar: Men, women and adolescents  16-20 visioning exercise: Unmarried adolescents  4 Key informant exercise: Women 16-20 risks and benefits: Fathers and mothers  Nepal: unknown # social mapping: Men and women 16 timelines: Fathers and mothers 40 visioning exercise: Unmarried and married adolescents 16 risks and benefits: Mothers and adult male community figures | Social mapping, timelines, seasonal calendar, visioning exercise, KII, risk benefits 4-day sense making workshop for analysis, disaggregated by context, within theory of change | Vulnerability to, drivers of, and experiences of early marriage |
| Mahato (2016) | Causes and Consequences of Child Marriage: A Perspective | Nepal (Dhanush) | 5 NGO staff | Telephone interviews No analysis method or theoretical underpinnings | Causes and consequences of early marriage |
| Ali (2021) | Perceptions of women, their husbands and healthcare providers about anaemia in rural Pakistan: Findings from a qualitative exploratory study | Pakistan (Rural Sindh) | 10 FGD (8-15/group): with women of reproductive age and their husbands 10 KII: Healthcare providers (gynaecologists, medical doctors, midwives, or TBAs) | FGD, KII Coding framework developed, no theoretical underpinnings | Knowledge of anaemia and strategies for prevention |
| Ehsan (2021) | Domestic violence against pregnant women and its effects on their reproductive health | Pakistan (Multan) | 16 IDI: Pregnant women who were physically abused, or previously pregnant women who miscarried due to physical violence | IDI Grouping inductive and deductive codes to code clusters, no theoretical underpinnings | Causes and effects of domestic violence |
| Wyatt (2021) | Predictors and occurrence of antenatal depressive symptoms in Galle, Sri Lanka: a mixed-methods cross-sectional study | Sri Lanka (Bope Poddala, Galle) | 12 PHM | IDI Thematic analysis, no theoretical underpinnings | Prevalence and risk factors of antenatal depressive symptoms |
| Abbreviations: ANC; antenatal care, ANM; auxiliary nurse midwife, CCT; conditional cash transfers, CHW; community health worker, DPHO; district public health officer, EDM; ethnographic decision models, FCHV; female community health volunteer, FGD; focus group discussion, GBV; gender-based violence, IDI; in-depth interview, IPV; intimate partner violence, IUGR; intra-uterine growth restriction KII; key-informant interview, LWH; lady health worker, MIL; mother-in-law, MPHW; multi-purpose health worker, n.d.; no date, NGO; non-governmental organisation; PHM; public health midwife, SBA; skilled birth attendant, SRHR; sexual and reproductive health rights, SSI; semi-structured interviews, TBA: traditional birth attendant, y/o; year old, yr; year, unknown #; unknown number | | | | | |
