# Supplementary material for: “Can’t live willingly”: A thematic synthesis of qualitative evidence exploring how early marriage and early pregnancy affect experiences of pregnancy in South Asia
Source: PLOS Glob Public Health. 2023 Oct 23;3(10):e0002279. doi: 10.1371/journal.pgph.0002279 (PMC10593245; doi:10.1371/journal.pgph.0002279)
Supplement: S4 Appendix — (DOCX) [file pgph.0002279.s004.docx]

| S4 Appendix. Heat map of themes and subthemes, in which a darker colour representing a higher referenced theme, with supporting evidence | | |
| --- | --- | --- |
| Theme | Name | Evidence |
| Household hierarchies preventing women and girls from speaking up and being listened to | Low position within the household negatively affecting pregnancy | “I knew that my condition was very serious, and everyone kept on telling me to try having the baby at home. I was trying, and I knew I couldn’t try anymore, but the others didn’t understand how serious it was.” Mother, aged 17, rural Bangladesh, 2009 (Sikder, 2011) “I took whatever pills my husband bought for me. Now I move around myself and buy whichever I feel like.” Mother who married young, Bangladesh, 2014 (Barua, 2018) "I didn't want or wish. My husband said one baby has died; take another, so we did" Girl married aged 12, Bangladesh, 2013/14 (Ainul, 2015) “They [in-laws] said that I do not need to go [to ANC] again. I have to stay quiet at that time... I can’t say anything, they are senior to me” Teenage mother, Nepal, 2006 (Simkhada, 2011)  “My mother- in- law influenced my decision. She has the right to make decisions regarding my contraception and childbearing” Married adolescent, Nepal, 2019 (Sekine et al., 2021)  “Who will cook again? My stomach is full if my family's stomach is full. I don't get hungry” Woman with child aged ≤6 months, Nepal Terai, 2019 (Morrison et al., 2019) |
|  | Varying role of husbands | "In joint families… a man is considered besharam (shameless) if he exhibits an ‘excessive’ interest in his pregnant wife" Author interpretation, rural Pakistan, 2001 (Mumtaz & Salway, 2009) |
|  | Decisions being made collectively within the family | "Adolescents frequently brought health concerns to family, then decisions were made collectively about the best course of action; adolescents described varying degrees of ‘voice’ within the collective" Author, Urban Bangladesh, 2019 (Pike et al., 2021) |
|  | Mothers-in-law being the most important influence during pregnancy | "Who else would understand what I am going through better than her? That's why she decides it" Mother, aged 17, rural Nepal, 2014-15 (Shahabuddin et al., 2019) |
|  | Family advice conflicting with healthcare provider advice | “Family members are superstitious and tell, ‘this is nonsense what the doctor says. Follow what we say.’” Female nurse, aged 26, urban Bangladesh, 2014-15 (Shahabuddin et al., 2017) |
|  | Alignment in the interests of family and pregnant women affecting pregnancy experiences and outcomes | "My mother-in-law makes the decision (about what to eat). I ate first during my pregnancy. I ate whenever I felt hungry. I ate everything—green leafy vegetables, fish, meat." Mother, rural Nepal, 2019 (Morrison et al., 2021) |
|  | Families using the young age of women as a justification for them making decisions | You are of a young age, whereas we are elder and we know more" Aunt, aged 26, Urban Bangladesh, 2019 (Pike et al., 2021)  “Young brides were likened to small wild birds to be tamed, or parrots which if taken in and raised from a young age would naturally imitate what they were taught” Author, rural Bangladesh, 2002 (Schuler et al, 2006) |
|  | Daughter-in-law's complaints not being heeded even when seriously ill | "A local teacher described a daughter-in-law as being oppressed within a family to such an extent that her complaints are not heeded even when she is ill" Author, rural Nepal, 2015 (Paudel et al., 2018) |
| Increased threat of abuse for adolescents | Girls married early having an increased risk of abuse within the home | “Unwanted sex is also a symbol of love. It is a way to resolve the dispute between husband and wife.” Participant married aged 17, urban Pakistan, 2013 (Nasrullah et al., 2015) “He complained to his mother who told him to tie me up and have sexual activity with me anyway." Case study of women married age 14 years, urban Pakistan (Hamid et al., 2008)  “Even last night they beat me, and I am bleeding. I am four months pregnant and I am bleeding’ Pregnant women married aged <18 years, Nepal (Human Rights Watch, 2015) "“This is part of marriage—many men behave like this, but young women these days think only of themselves.” Older woman, urban Pakistan (Hussain & Khan, 2008) "[For] the younger participants who experienced frequent violence from early days of their marriage, violence intensiﬁed during pregnancy" Author, urban Pakistan (Hussain & Khan, 2008)  “Her husband agreed to hospitalization, but he threatened her with divorce if she divulged any information about his violent behavior to the hospital staff” Author, urban Pakistan (Hussain & Khan, 2008) |
|  | Disrespectful care from care providers being common among young women | "Young women having their first babies were particularly distressed but their cries for help, for their mothers or for God were largely ignored." Author on healthcare provider violence, urban Afghanistan, 2010-12, (Arnold et al., 2019) "The doctor shouted at me it is not only you who give birth to a baby, you elope at the age of 17 and you women are looking for your rights." Girl married aged 17 years, rural Nepal, 2015 (Maharjan et al, 2019)  "I was left alone in the stirrups legs up. I was screaming and nobody came." Teenage mother, urban Afghanistan, 1996 (Kaartinen & Diwan, 2002) "“They had already scolded me for first pregnancy at very young age. I was furious at that time. If I had gone for the check-up for second pregnancy, they would even scold me very badly. That is why I didn't go for a check-up." Girl married aged 14 years, rural Nepal, 2015 (Maharjan et al, 2019) |
|  | Marital instability increasing anxiety during pregnancy | "So I am all alone. I don't have any place to go and express my feelings and he (husband) is everything I have so, I tolerate him and I don't have any other alternatives (tears in her eyes)" Pregnant girl aged 17 years, urban Nepal, 2013 (Debua et al., 2016) |
|  | Young age increasing physical vulnerability to violence | “I was young and weak, I opposed him, he used to beat me, stopped giving me food, even tortured me during my periods… I wish I was never born” Woman married age 13 years, rural India (Mukherjee, 2018) “One needs to be strong to endure a beating, don’t you know that? People should marry their daughters after they get a bit older.’’ Mother, rural Bangladesh, 2002 (Schuler et al, 2006) |
| Early marriage affecting knowledge of pregnancy needs | Early marriage limiting education which impacts pregnancy | "Who would do it if one knew everything? If I had known everything, I would not be pregnant" Mother, aged 17, rural Nepal, 2015 (Maharjan et al, 2019)  “At home, I was in labor pain for three days. It contracts and leaves. I thought the date had not yet approached. That’s why I didn’t even tell my husband.” Woman who had high risk pregnancy, Nepal, 2019-2020 (Rajbanshi et al., 2021)  “Depending on the tradition, certain foods were avoided during pregnancy including coconut, pineapple, papaya, nuts, some kinds of fish and meat and (more rarely) categories like ‘non-liquid foods’ or ‘cold foods.’ Mainly, it was believed that these foods could harm the baby” Author, rural Bangladesh, 2019 (Pike et al, 2021)  “I used to enjoy student life, without studies, life is very difficult.” Girl married aged 14, rural Bangladesh, 2014 (Human Rights Watch, 2015) |
|  | Families valuing experience over learned knowledge | “Authoritative knowledge in this environment is not based on the biomedical understanding of reproductive health; Rather, it is based on women’s practices over generations” Woman, rural Pakistan, 2013 (Sultana et al., 2022)  "Another woman had no idea what to expect by the time she was married because she had no sisters to explain to her about married life" Author, Pakistan, 1992-93 (Winkvist & Akhtar, 2000)  "Women say, ‘haven't we gone through these days? We did not need special care back then.’ Nowadays new doctors have come, new ideas have come." FCHV aged 23, urban Bangladesh, 2019 (Pike et al, 2021) |
|  | Adolescents needing support from family to learn due to young age | "Because she's young so she can't learn. She is a child herself that's why we've given her instructions. She has learned" Aunt, aged 35, Urban Bangladesh, 2019 (Pike et al., 2021) "Those who are underage … we understand less, or, we do not have the ability to understand" Pregnant girl aged 15, rural Bangladesh, 2019 (Pike et al, 2021) |
|  | Contradiction! Knowledge not helping help girls | "I understand the importance of doctors. I wanted to deliver my first child in the hospital but my mother-in-law did not allow me to go" Girl married aged 13, rural India (Mukherjee, 2018) |
| Pregnancy being a time of shame | Participants considering pregnancy as a shameful time | "She has made her body a space of uncontestable honor for her parents, and... she is expected to act in a contradictory way. It is now honorable to allow access to her body by her husband. In the confused shame of ambiguity, the pregnant woman avoids telling others." Author, rural Nepal, 1988/89 (Cameron, 1993) “I will not allow my daughter-in-law to go outside of the locality. Community people will say bad things about my newly married daughter-in-law and about the family, so I can’t let that happen.” Influential female, rural Bangladesh, 2017 (Samandari et al, 2020) “Pregnancy, an obvious manifestation of sexual activity, is associated with ‘sharam’ (shame). Pregnant women should avoid public space” Author, rural Pakistan, 2001 (Mumtaz & Salway, 2007) |
|  | Pregnancy being a time of emotional stress | "Psychological impact was reported in terms of depression, unexplained anxiety, and lack of confidence and over dependence." Author, rural India, (Mitra &  Parasuraman, 2015)  "From the mothers I have met, teenage mothers... have a greater tendency to develop depression/anxiety" Midwife, Sri Lanka, 2020 (Wyatt et al., 2021)  "I was married off at a very early age, brought into a new family, I had to adjust with a new family and with my husband. Everything was haphazard and in the middle of all those changes I became a mother too. It was all very overwhelming" Girl married age 12, Bangladesh, 2013/14 (Ainul, 2015)  “Rashi recounted feeling scared, helpless, anxious and unable to share her pain or seek help from anyone.” Author, urban India, 2015-2016 (Sama, 2019) |
|  | Shyness among younger women affecting care seeking | “I was avoiding [seeking care]. There was a feeling of shyness within me.” Adolescent married age 17, rural Nepal, 2015 (Maharjan et al., 2019) “Older women are going to health-post easily after delivery also, but these early-married women are very shy to go.” NGO worker, rural Nepal, 2015 (Maharjan et al., 2019) "Her young daughter-in-law was very embarrassed about her body image in her pregnancy and was not eager to go for an ANC check-up. She believed that physical appearance was more important to some teenage women and can be a barrier for such women to attend ANC" Author, Nepal, 2006 (Simkhada, 2011) “[Early married women] go with one problem, and they say they have a headache, which is easier for them to tell and bring the wrong medicine." NGO worker, rural Nepal, 2015 (Maharjan et al., 2019) |
| Girls being pressured into pregnancy to earn social position within their household | Girls needing to earn social position through marriage and pregnancy | “Married adolescent girls… realised that social acceptance and security in the marital home were established largely through fertility, particularly the birth of a son” Author, rural Nepal (Matsuyama, 2002)  ‘Shohagi,** aged 14, had a love marriage and a baby was the only way she could improve her relationship with both her husband, who wanted a child, and her in-laws, who did not accept her… “If you have a child, sister, you can exercise your rights but if you don’t have a child then you have no rights.’’ Author & Married girl aged 14, urban Bangladesh, 2001-03 (Rashid, 2006) ‘Such experiences were also recounted by other women and that it had caused them sadness and anxiety due to the immense social and familial pressure on them to prove their fertility and produce children soon after marriage.’ Author, urban India, 2015-2016 (Sama, 2019) "If he gets upset he starts beating me. I hope I have a son so that I am respected." Case study, Hamid "I am not able to give him a son, I have only given birth to daughters during the 3 years. I have 3 daughters already and this one is also a daughter (pointing out abdomen). We went for sex identification one month ago and came to know that this one is also a daughter. Since that day, he (husband) started drinking in unlimited amounts. He comes home at midnight and beats me with such a big stick (showing the size with her hand)." Pregnant girl aged 17 years, urban Nepal, 2013 (Debua et al., 2016) "Asked why she got pregnant repeatedly at such a young age, she said, “I know it is not good for my health. But my parents-in-law said to me that I should produce at least one child. Otherwise, they would bring a co-wife to my husband Author & Woman who lost baby aged 15, rural Nepal (Matsuyama, 2002)  “They actually send their daughter-in-laws back to her parents and search for a new bride for their sons to that they can bare them sons.” Woman aged 30, rural India, 2011 (Roberts et al., 2012) |
|  | Community beliefs and opinions influencing marital and family decisions | "Gharbar is a gendered construction by which it is perceived that a girl is secured and settled when she is married, gives birth early and has a surviving child, preferably a baby boy... The parents are influenced by everyone’s concerns including relatives, neighbours and peers." Author, rural Nepal, 2015 (Paudel et al., 2018) “My neighbours would say to my husband, ‘you have a son now. She has given birth to a child so why do you let them give her so much of a hard time?’” Adolescent mother, urban Bangladesh, 2001-03 (Rashid, 2011) |
|  | Families having distrust of newly married women and girls | "My mother-in-law says I am not innocent and my husband says that I have to learn to be obedient and submissive" Case study of woman married age 14 years, urban Pakistan (Hamid et al., 2008)  “When she tells about her illness, the mother-in-law often does not trust; mother-in-law thinks that she is telling a lie (Nauragareko)” Teacher, rural Nepal, 2015 (Paudel et al., 2018)  "She would be able to hold on to my son. If she gave him a baby boy, he would listen to her and she could do what she wanted...’’ Mother-in-law of adolescent, urban Bangladesh, 2001-03 (Rashid, 2006) |
| Risk being normalised among younger pregnant women | Young age making girls clinically vulnerable | "All the [young] women were anaemic" Author, urban India, 2020-21 (Khanna et al., 2022)  "Reproductive organs in teenagers are not matured enough, so there is a high chance of premature birth and underweight babies being born. Moreover if they do not get special care during delivery they may die" Care taker of adolescent mothers, urban Nepal, 2015 (Bhandari & Joshi, 2016) "Adolescent patients often suffer from “pre-term labour, waters breaking early, prolonged and obstructed labour, pulmonary hypertension, restricted growth of the foetus in the uterus, anaemia, intractable nausea, vomiting and dehydration, genital injuries, malnourishment, and psychological trauma”’ Doctor supervising maternal health care program, Bangladesh, 2014 (Human Rights Watch, 2015) |
|  | Care only being sought in severe cases | "As I had a normal delivery, I never visited the doctor because there were no complications.” Young married woman, urban India, 2020-21 (Khanna et al., 2022)  "they’re brought to us with pregnancy complications when it’s difficult to save their lives" Physician, Pakistan, 2017-18 (Omer et al., 2021) |
|  | Early marriage being considered a cause of adverse outcomes | “It is because you married her early that she is having these pregnancy-related problems” Health worker, Nepal (Human Rights Watch, 2015)  "Health problems among married girls were attributed to their subjection to sex at young ages" Author, rural Bangladesh, 2002 (Schuler et al, 2006) |
|  | Normalisation and expectation of risk among young pregnant women causing fear | "[Young pregnant woman]: ‘Will [my baby] live or die? Nobody knows.’  [Interviewer]: ‘You are afraid.’  [Young pregnant woman]: ‘Yes’ " Rural Nepal, 1988/89 (Cameron, 1993) "I was very afraid during delivery because I thought I would die… One of my friend expired during delivery at the age of 16…" Young mother, urban Nepal, 2015 (Bhandari & Joshi, 2016) |
|  | Over-medicalisation in hospitals preventing care-seeking | "Early childbearing with the need for emergency caesarian deliveries which they saw as prohibitively expensive and feared might cause permanent weakness" Author, rural Bangladesh, 2002 (Schuler et al, 2006)  “There are a lot of problems if you go to the hospital, if you do a C-section there is a problem in moving. There is a problem in eating, for three to four months you can’t do any heavy work” Pregnant adolescent aged 17, rural Bangladesh, 2014-15 (Shahabuddin et al., 2017) |
|  | Contradiction! Young age reducing risk during pregnancy | "In those days, we had innocent women who were younger as our clients. Nowadays women get pregnant much later. This leads to complications. Not only this, the women and their families are well informed. They know everything and ask a lot of questions. Because of this, we are seeing a lot of complications” Community Health Worker, urban India, (Gopichandran et al., 2018) |
| Girls feeling isolated during pregnancy | Younger women feeling isolated and lonely during pregnancy | "From the beginning, I just stay at home. I don’t wander around, so how may I know [anyone]? (laughs)" Newly married woman, Nepal, 2019-2020 (Rajbanshi et al., 2021) “I am feeling lonely and helpless.... I am so anxious about it. I am feeling sick. I have fear. I am worried.” Pregnant girl aged 17, rural Nepal, 2010 (Kaphle et al., 2013) "I was able to live independently. Now I walk less, hang around less; I have to sit at home, can't go anywhere outside. Can't live willingly." Pregnant girl aged 18, urban Bangladesh, 2019 (Pike et al, 2021) "Due to the huge age gap between husband and wife there is difference in mutual understanding, result[ing in] the girls [feeling] rejected, isolated, and depressed" Author, Nepal, (Mahato, 2016) |
|  | Community being beneficial for pregnant women | "I had a pregnant neighbour with close delivery dates. We would go together" Mother aged 17, rural Nepal, 2014-15 (Shahabuddin et al., 2019) "We have a women's group and we sit and have a discussion. I came to know about [the importance of ANC visits and delivery care] from the group" Mother aged 15, rural Nepal, 2014-15 (Shahabuddin et al., 2019) "So, whenever we have a big argument I visit my maternal home…" Pregnant girl aged 19, urban Nepal, 2013 (Debua et al., 2016) "Another woman had no idea what to expect by the time she was married because she had no sisters to explain to her about married life" Author, Pakistan, 1992-93 (Winkvist & Akhtar, 2000) |
|  | Young pregnant women having restricted movement during pregnancy | "Proscriptions against women moving outside…, particularly by younger women, in order to avoid danger of loss of izzat (male/family honour)" Author, rural Pakistan, 2001 (Mumtaz & Salway, 2007) "I do not go anywhere, even places near my house, because my mother-in-law suspects that if I talk to anybody, I might be badly influenced by them.” Married girl aged 18, rural Bangladesh, 2017 (Samandari et al, 2020)  “Restrictions on [the] movement of younger women, lack of access to household money and cultural norms around respect and positionality in the household prevented them from making decisions or buying food.” Author, rural Nepal, 2019 (Morrison et al., 2021) |
| Conflicting reproductive & domestic or income-generation roles | Increased workload putting pregnancy at risk | "Who would do the household work or take care, and then they didn’t let me go [to ANC]. So I stayed home" Woman with experience of neonatal death aged 18, rural Bangladesh, 2014-15 (Shahabuddin et al, 2017) "Pregnant women do every kind of work. The harder your work during pregnancy, the easier it will be for delivery" Husband, rural Nepal (Matsuyama, 2002)  "She had gone to bring fodder from the forest. She slipped on the road, hurt her abdomen. She had bleeding and fainted… Fortunately, the mother survived; however, the baby died" Lady health worker, rural Nepal, 2015 (Paudel et al., 2018) “I am really ashamed at forcing such a very young girl to do heavy chores, but what is the alternative? What is the point [of having her here] if I spare her?" Woman with daughter-in-law aged 14, rural Bangladesh, 2002 (Schuler et al, 2006)  "Pregnant women do every kind of work. The harder your work during pregnancy, the easier it will be for delivery" Husband, rural Nepal (Matsuyama, 2002) |
|  | Women feeling pressured to choose between having a family or income-generating work | "[My husband] really wanted a baby. I was working in the garments. I wanted to work for longer, save up some money and then have a child... My mother convinced me to have a baby to make him happy.’’ Girl married aged 14, urban Bangladesh, 2001-03 (Rashid, 2006)  "My father-in-law wants me to work and bring money for the household. If I fall pregnant I cannot work, can I?" Girl aged 17 who was forced into an abortion, urban Bangladesh, 2001-03 (Rashid, 2006) |
|  | Pregnancy complications preventing income-generation and/or domestic work after birth | “You know, I don't want to hide this. I have prolapsed uterus (Aangjhadne) [during this young daughter's birth]. If I carry any load of firewood or grass or any heavy thing, I feel it coming out, bulging out. It is burning and painful.” Young mother, rural Nepal, 2015 (Paudel et al., 2018) |
|  | Social benefits of women working outside of the home | ““If my daughter gets a job or continues working as a tailor,’’ she explained, ‘‘then her parents-in-law will not challenge her even if she fails to do the family chores… My daughter will carry some weight in her family.”” Mother, rural Bangladesh, 2002 (Schuler et al., 2006) |
| Early marriage intersecting with other identities | Poverty affecting experiences of pregnancy | "Mental stress due to an unpaid loan, hard physical labour and poor nutrition during pregnancy all took their toll and resulted in a neonatal death" Author, urban India, 2020-21 (Khanna et al., 2022) "If we have money, we can take her to medical. If we don't have money, we stay at home and pray to Allah" Mother-in-law, urban Bangladesh, 2019 (Pike et al, 2021)  "The doctors tell us to take these in full stomach. But we are poor people. We cannot have full stomach always … due to financial problems" Pregnant adolescent, urban Bangladesh, 2019 (Pike et al, 2021) "I wanted to call the doctor. I was so sad that my husband said we should wait longer. I was trying so hard. I didn’t want to go through so much pain just so we wouldn’t have to spend money.” Pregnant adolescent aged 16, rural Bangladesh, 2009 (Sikder, 2011) |
|  | Caste group affecting experiences of pregnancy | "Women faced restrictions on their movement, particularly young or Muslim women, and women from conservative or ‘high-class’ families: ‘My husband doesn't let me go outside that often'" Author/woman, rural Nepal, 2019 (Morrison et al., 2021) "The [women] from a poor background like Dalits, Janajati, are treated differently. On top ofthat, adolescents are more vulnerable; they are humiliated and treated differently.” District officer, Dang Nepal, 2015 (Maharjan et al., 2019) "The evidence is convincing that lower caste women have more decision-making power and control in their marriages and family lives than do upper caste women because of their greater economic autonomy in the household" Author, rural Nepal, 1988/89 (Cameron, 1993) |
|  | Contradiction! Poverty not being a cause of adverse outcomes | "A young girl experiencing severe difficulties during her first delivery was left to die because the family's male members refused to spend the money and time to transport her to a hospital..., saying that "We do not have the money to cover the expenses", even after the local men had offered to carry her there for free." Author (on father of adolescent mother), rural Nepal, 1988/89 (Cameron, 1993) "I would not say poverty is the main reason... Here, they are offered to get treatment almost for free’ Gynecologist, rural Bangladesh, 2014-15 (Shahabuddin et al., 2017) |
|  | Religious practices affecting reproductive health practices | "My service area is a Muslim community and the major challenge that they face is poor spacing between children an0d having many kids to deal with.” Midwife, Sri Lanka, 2020 (Wyatt et al., 2021) |
