# Supplementary material for: “Can’t live willingly”: A thematic synthesis of qualitative evidence exploring how early marriage and early pregnancy affect experiences of pregnancy in South Asia
Source: PLOS Glob Public Health. 2023 Oct 23;3(10):e0002279. doi: 10.1371/journal.pgph.0002279 (PMC10593245; doi:10.1371/journal.pgph.0002279)
Supplement: Attachment — Submitted filename: Response to editor.docx [file pgph.0002279.s006.docx]

University College London

30 Guilford Street

London, UK

WC1N 1EH

3^rd^ August 2023

Dear Oliver Ian Tolentino

We thank you for your time and careful consideration of our manuscript PGPH-D-23-00724 entitled ““Can’t live willingly”: A thematic synthesis of qualitative evidence exploring how early marriage and early pregnancy affect experiences of pregnancy in South Asia”. We are grateful for the opportunity to address the issues you have identified.

The comments provided are shown, with our responses below. We have also included a manuscript with ‘tracked changes’ on.

Issues:

1. Please amend your online detailed Financial Disclosure statement. This is published with the article. It must therefore be completed in full sentences and contain the exact wording you wish to be published.

a) State the initials, alongside each funding source, of each author to receive each grant. For example: "This work was supported by the National Institutes of Health (####### to AM; ###### to CJ) and the National Science Foundation (###### to AM)."
b) State what role the funders took in the study. If the funders had no role in your study, please state: “The funders had no role in study design, data collection and analysis, decision to publish, or preparation of the manuscript.”"

Response: We have updated the financial disclosure in the provided format.

2. Please update your online Competing Interests statement. If you have no competing interests to declare, please state: “The authors have declared that no competing interests exist.”

Response: We have updated the competing interests statement in the provided format.

3. Please ensure that you refer to Figure 3 in your text as, if accepted, production will need this reference to link the reader to the figure.

Response: We have corrected the references to figure 3 in the text.

4. We noticed that you used “Unpubl Manuscr” in the manuscript. We do not allow these references, as the PLOS data access policy requires that all data be either published with the manuscript or made available in a publicly accessible database. Please amend the supplementary material to include the referenced data or remove the references.

Response: We have updated the references as these have both been published since submission.

5. Figures 3 and 4: Please confirm whether you drew the images / clip-art within the figure panels by hand. If you did not draw the images, please provide (a) a link to the source of the images or icons and their license / terms of use; or (b) written permission from the copyright holder to publish the images or icons under our CC-BY 4.0 license. Alternatively, you may replace the images with open source alternatives. See these open source resources you may use to replace images / clip-art:

Response: We have replaced the icons with those from openclipart.org/, which are open access. We have included the source in the figure legend. We have removed the icons from figure 4.

Figure 3: please (a) provide a direct link to the base layer of the map (i.e., the country or region border shape) and ensure this is also included in the figure legend; and (b) provide a link to the terms of use / license information for the base layer image or shapefile. We cannot publish proprietary or copyrighted maps (e.g. Google Maps, Mapquest) and the terms of use for your map base layer must be compatible with our CC-BY 4.0 license.

Response: The map in figure 3 was obtained from datawrapper.de. As stated on their website (<https://www.datawrapper.de/faq#can-i-use-my-charts-or-maps-in-a-commercial-product-or-publication>), the user (Faith Miller) has the full copyright to the map. We have included the label in the map which was mistakenly edited out and have included the source in the figure legend.

We hope that you will be satisfied with the amendments made. If there are any further issues do not hesitate to get in touch. We would like to thank you again for your time and consideration of our manuscript.

Yours Sincerely,

Faith Miller
